# Clinical biomarker-based biological aging and risk of cancer in the UK Biobank

**DOI:** 10.1038/s41416-023-02288-w

**Published:** 2023-04-29

**Authors:** Jonathan K. L. Mak, Christopher E. McMurran, Ralf Kuja-Halkola, Per Hall, Kamila Czene, Juulia Jylhävä, Sara Hägg

**Affiliations:** 1 Department of Medical Epidemiology and Biostatistics, Karolinska Institutet, Stockholm, Sweden; 2 Department of Clinical Neurosciences, University of Cambridge, Cambridge, UK; 3 Department of Oncology, Södersjukhuset, Stockholm, Sweden; 4 Faculty of Social Sciences (Health Sciences) and Gerontology Research Center (GEREC), University of Tampere, Tampere, Finland

**Keywords:** Cancer epidemiology, Epidemiology, Biomarkers

## Abstract

**Background:**

Despite a clear link between aging and cancer, there has been inconclusive evidence on how biological age (BA) may be associated with cancer incidence.

**Methods:**

We studied 308,156 UK Biobank participants with no history of cancer at enrolment. Using 18 age-associated clinical biomarkers, we computed three BA measures (Klemera-Doubal method [KDM], PhenoAge, homeostatic dysregulation [HD]) and assessed their associations with incidence of any cancer and five common cancers (breast, prostate, lung, colorectal, and melanoma) using Cox proportional-hazards models.

**Results:**

A total of 35,426 incident cancers were documented during a median follow-up of 10.9 years. Adjusting for common cancer risk factors, 1-standard deviation (SD) increment in the age-adjusted KDM (hazard ratio = 1.04, 95% confidence interval = 1.03–1.05), age-adjusted PhenoAge (1.09, 1.07–1.10), and HD (1.02, 1.01–1.03) was significantly associated with a higher risk of any cancer. All BA measures were also associated with increased risks of lung and colorectal cancers, but only PhenoAge was associated with breast cancer risk. Furthermore, we observed an inverse association between BA measures and prostate cancer, although it was attenuated after removing glycated hemoglobin and serum glucose from the BA algorithms.

**Conclusions:**

Advanced BA quantified by clinical biomarkers is associated with increased risks of any cancer, lung cancer, and colorectal cancer.

## Background

Aging is closely linked to cancer [1–3], in which some of the proposed hallmarks of aging, such as genomic instability, cellular senescence, and epigenetic alteration [4], also overlap with the hallmarks of cancer [5]. Although chronological age (CA) is the dominant risk factor for most cancers [6], it does not capture the heterogeneity between older individuals. Biological age (BA), on the other hand, combines information from biological markers and may better reflect an individual’s physiology and risks of age-related diseases and death [7].

In recent years, various BA measures have been proposed and validated, including telomere length, deficit-accumulation frailty indices, epigenetic clocks based on DNA methylation markers, and algorithms that combine information on multiple clinical biomarkers [7, 8]. Accumulating evidence from observational studies has suggested that epigenetic clocks may predict cancer risks [9–11]. Recently, a Mendelian randomization study provided further support on the potential causal relationship between GrimAge acceleration, a second-generation epigenetic clock that reflects not only CA but also mortality and smoking [12], and the risk of colorectal cancer [13]. On the other hand, various biomarkers from blood chemistries (e.g., total cholesterol [14], glucose [15], C-reactive protein [16]) and other clinical data (e.g., waist circumference [17], forced expiratory volume [FEV_1_] [18]) have been linked to cancer risks. However, there has been a lack of data on whether composite measures of BA based on these routinely collected clinical biomarkers may predict cancer risks. As different types of BA measures may capture slightly different aspects of the aging process [19, 20], deciphering the link between various BA measures and cancers is important for understanding the mechanisms underlying aging and cancer.

Therefore, the aim of this study was to investigate the relationships between BA quantified based on clinical biomarkers and the risk of any cancer and site-specific cancers (including breast, prostate, lung, colorectal, and melanoma skin cancer), using data from the large population-based UK Biobank cohort. We hypothesize that these composite biomarker measures, as proxies for biological aging capturing the overall aging process, would be associated with increased risks of cancers independent of CA and other risk factors.

## Methods

### Study population

During 2006–2010, the UK Biobank enrolled over 500,000 participants aged 37–73 years from the general population [21]. At baseline, participants completed a touch-screen questionnaire, provided biological samples, and had physical measurements taken in 22 assessment centers throughout England, Wales, and Scotland. The UK Biobank study was approved by the North West Multi-Centre Research Ethics Committee. All participants provided written informed consent.

In this analysis, we included 331,699 UK Biobank participants who had complete data on the 18 biomarkers used in our BA algorithms (Table 1). We also excluded *n* = 177 with outlier BA values (considered as ±5 standard deviations [SD] from mean) and *n* = 23,366 with any cancer diagnosis (except non-melanoma skin cancer) before baseline, yielding an analytical sample of *n* = 308,156.

**Table 1:** Biomarkers included in the biological age algorithms.

Biomarkers available in NHANES III and UK Biobank^a^	Correlation with CA in NHANES III	Included in new algorithms of KDM, PhenoAge, and HD^b^	Included in Levine original KDM^c^	Included in Levine original PhenoAge^c^
FEV_1_ (L)	−0.62	Yes	Yes	—
Pulse rate (beats/min)	0.61	—	—	—
SBP (mm Hg)	0.59	Yes	Yes	—
Blood urea nitrogen (mg/dL)	0.46	Yes	Yes	—
HbA1c (%)	0.32	Yes	Yes	—
Total cholesterol (mg/dL)	0.32	Yes	Yes	—
Creatinine (μmol/L)	0.30	Yes	Yes	Yes
Serum glucose (mmol/L)	0.26	Yes	—	Yes
Waist circumference (cm)	0.26	Yes	—	—
Red cell distribution width (%)	0.23	Yes	—	Yes
Albumin (g/dL)	−0.22	Yes	Yes	Yes
Alkaline phosphatase (U/L)	0.21	Yes	Yes	Yes
Triglyceride (mg/dL)	0.18	Yes	—	—
Mean cell volume (fL)	0.17	Yes	—	Yes
Uric acid (mg/dL)	0.16	Yes	—	—
Lymphocyte (%)	−0.14	Yes	—	Yes
RBC count (million cells/μL)	−0.14	Yes	—	—
C-reactive protein (mg/dL)	0.11	Yes	Yes	Yes
DBP (mm Hg)	0.11	Yes	—	—
Monocyte (%)	0.06	—	—	—
Total bilirubin (mg/dL)	−0.03	—	—	—
BMI (kg/m^2^)	0.02	—	—	—
WBC count (1000 cells/μL)	−0.02	—	—	Yes
HDL (mg/dL)	0.00	—	—	—

*BMI* body mass index, *CA* chronological age, *DBP* diastolic blood pressure, *FEV*_*1*_ forced expiratory volume in 1 second, *HbA1c* glycated hemoglobin, *HD* homeostatic dysregulation, *HDL* high-density lipoproteins, *KDM* Klemera-Doubal method, *NHANES* National Health and Nutrition Examination Survey*, RBC* red blood cell, *SBP* systolic blood pressure, *WBC* white blood cell.

^a^Only biomarkers that were available in UK Biobank and had ≤20% missing data in NHANES III (i.e., training set) were considered.

^b^We included 18 biomarkers which were correlated with chronological age (|*r*| > 0.1) in the new algorithms of KDM, PhenoAge, and HD. Pulse was not included because it had high correlation with systolic blood pressure (*r* = 0.84) (i.e., the models did not converge when including it). All algorithms were parametrized in NHANES III using the R package *BioAge* [25]. As a sensitivity analysis, we also created modified versions of the KDM, PhenoAge, and HD after excluding HbA1c and serum glucose from the algorithms (see Supplementary Table 6).

^c^For comparison of the new algorithms, we used the original list of biomarkers included in Levine 2013 (except cytomegalovirus optical density which was not available in UK Biobank) [28] and Levine et al. [23] to calculate the “Levine original KDM” and “Levine original PhenoAge”, respectively.

### Biological age measures

We quantified BA based on three composite measures of blood chemistry and other clinical data: Klemera-Doubal method (KDM) [22], PhenoAge [23], and homeostatic dysregulation (HD) [24]. Details on the calculations and interpretations of the three measures have been summarized previously [25, 26]. Briefly, KDM is calculated from a series of regressions of biomarkers on CA and can be interpreted as the age at which the average physiology in the US National Health and Nutrition Examination Surveys (NHANES) III (i.e., the training sample) matches the physiology of the person. PhenoAge is calculated based on a mortality prediction score of biomarkers and CA and can be interpreted as the age at which the average mortality risk in NHANES III matches the predicted mortality risk. Different from KDM and PhenoAge, HD does not include CA in the calculation, but it is calculated based on the Mahalanobis distance [27] for a set of biomarkers relative to a reference sample and can be interpreted as the deviation of the person’s physiology from a healthy sample of NHANES III participants aged 20–30. All three measures have previously been validated for their abilities to predict diseases, disability, and mortality [25, 26].

In general, any age-related biomarkers can be used for constructing the BA algorithms. To facilitate comparison, we selected the same set of biomarkers in our KDM, PhenoAge, and HD algorithms, which were computed using the R package *BioAge* [25] in three steps:

1. Training in NHANES III. We first identified 19 potential biomarkers covering a range of organ systems (e.g., cardiometabolic, inflammatory, kidney, lung functions) that are routinely collected in clinical practice and were available in NHANES III and UK Biobank (Supplementary Fig. 1). Only those with ≤20% missing data and correlated with CA ( |*r* | > 0.1, in accordance with prior work [19, 28]) in NHANES III were considered. Pulse was excluded due to its high correlation with systolic blood pressure (*r* = 0.84). Therefore, our new KDM, PhenoAge, and HD algorithms included 18 biomarkers (Table 1): FEV_1_, systolic blood pressure, blood urea nitrogen, glycated hemoglobin (HbA1c), total cholesterol, creatinine, serum glucose, waist circumference, red cell distribution width, albumin, alkaline phosphatase, triglyceride, mean cell volume, uric acid, lymphocyte percent, red blood cell count, C-reactive protein, and diastolic blood pressure. Following previous work [25], we selected non-pregnant participants who aged 30–75 years and had complete biomarker data as the reference population for KDM (*n* = 7694). The reference population for PhenoAge included those aged 20–84 years and with complete biomarker data (*n* = 12,998). The reference population for HD included participants aged 20–30 years who were not obese and whose biomarker values were within the age- and sex-specific normal range (*n* = 258). Only one measurement occasion was available per person in the training set.
2. Testing the new BA algorithms in comparison to the published algorithms for their ability to predict mortality in an independent cohort of NHANES IV participants (*n* = 3849), who were recruited during 1999–2014 and followed up to 2015. Similar to the original version of KDM [28] and PhenoAge [23] (constructed using an alternative list of biomarkers as shown in Table 1), our new BA algorithms were statistically significantly associated with mortality during a median follow-up time of 7.4 years (interquartile range 4.1–11.5) (Supplementary Table 1). Besides, the new KDM and PhenoAge were strongly correlated with CA (*r* > 0.9), and all BA measures were moderately correlated with each other in NHANES IV (Supplementary Figs. 2 and 3).
3. Projecting the newly trained algorithms onto UK Biobank. Correlations among the 18 biomarkers are shown in Supplementary Table 2, and the distributions of the BA measures in UK Biobank are presented in Supplementary Fig. 4.

To calculate the deviation between BA and CA, we regressed out CA (as 3 degrees-of-freedom natural spline) from KDM and PhenoAge in each cohort and considered them as “age residuals” (also known as “age acceleration”) [29]. Residuals were not calculated for HD as it was not an age measure by definition and it already quantifies deviation from a reference population [25, 26], but it was log-transformed due to the skewed distribution. Higher values of KDM residual, PhenoAge residual, and HD represent advanced BA. The KDM residual, PhenoAge residual, and HD were then standardized with mean = 0 and SD = 1 to allow comparison of effect sizes in subsequent analyses.

### Cancer ascertainment

We studied five common cancers in Europe, including breast (for women), prostate (for men), and for both sexes, lung, colorectal, and melanoma skin cancer [30]. Incident cancers were ascertained from the cancer registries in England, Wales, and Scotland, where complete follow-up was available through February 29, 2020. We defined cancer diagnoses using the International Classification of Diseases, 10th revision (ICD-10) codes: any cancer (C00-97, excluding non-melanoma skin cancer C44), breast (C50), prostate (C61), lung (including trachea, C33-34), colorectal (C18-20), and melanoma (C43).

### Statistical analyses

Participants were followed from the date of baseline assessment to the date of cancer diagnosis, death, or end of follow-up, whichever occurred first. Hazard ratios (HRs) for cancer risks per 1-SD increase in each BA measure were estimated using multivariable Cox proportional-hazards models, where attained age was used as the underlying timescale. The models were first adjusted for birth year and sex, and were further adjusted for baseline assessment center, ethnic background, body mass index (BMI), smoking, alcohol, physical activity, education, and Townsend deprivation index [31] in analyses of all cancer sites. Additionally, we adjusted for cancer-specific covariates such as family history of cancers, women-specific factors (menopausal status, hormone replacement therapy use, oral contraceptive use, parity), cancer screening, diet, and sun exposure variables, as relevant for each cancer site based on the literature. Covariates used in each model are listed in the footnote of the corresponding tables, and their definitions and descriptive statistics are provided in Supplementary Table 3. Missing data on covariates were coded as indicator variables in the models. To assess the associations of individual clinical biomarkers—in comparison to composite BA measures—with cancer risks, we also calculated the HRs per 1-SD increase in each clinical biomarker from the fully-adjusted models.

We performed subgroup analyses to test whether the associations may differ by age at baseline (<60 vs. ≥60 years), sex (women vs. men), and ethnicity (white vs. non-white). For breast cancer and lung cancer, we additionally stratified the analyses by menopausal status (premenopausal vs. postmenopausal) and smoking (never-smokers vs. ever-smokers), respectively. The proportional-hazards assumption was formally tested using Schoenfeld residuals. When the proportional-hazards assumption did not hold in the exposure of interest (*P* < 0.05), we fitted a time-varying model by including interaction terms between the BA measure and age (split into 5-year intervals) to calculate HRs over different periods of follow-up. Besides, to examine potential non-linear relationships between BA measures and cancer risks, we compared model fit of a restricted cubic spline model with a linear model using likelihood ratio tests and plotted the models with evidence of non-linearity (*P* < 0.05).

Several sensitivity analyses were performed. First, we assessed the association between the original KDM [28] and PhenoAge [23] algorithms (Table 1) and cancer risks to analyze if the biomarker composition would affect the results. Second, as we found an unexpected protective effect of BA measures for prostate cancer and that HbA1c and serum glucose may be associated with reduced prostate cancer risk [32, 33], we repeated the analysis using modified versions of KDM, PhenoAge, and HD computed from 16 biomarkers (i.e., removing HbA1c and serum glucose) to further examine whether the observed inverse relationship may be influenced by these two biomarkers. Third, instead of using indicator variables for missing values, we performed a complete-case analysis using available data (i.e., excluding individuals with missing data on any covariates). Finally, as individuals with a cancer diagnosis during the first 2 years of follow-up might have undiagnosed or subclinical disease at baseline, we performed a sensitivity analysis by excluding the first 2 years of follow-up to minimize reverse causation.

All analyses were performed in R 4.1.3 and Stata 16. To account for multiple testing (3 BA measures × 5 cancers), we applied the Bonferroni correction and considered a two-sided *P* < 0.0033 (i.e., 0.05/15) as statistically significant.

## Results

### Sample characteristics

Of the 308,156 UK Biobank participants, the mean age at baseline was 56.2 years (SD 8.1) and 163,022 (52.9%) were women (Table 2). During a median follow-up of 10.9 years (interquartile range 10.1–11.6), a total of 16,933 (10.4%) and 18,493 (12.7%) incident cancers were reported in women and men, respectively.

**Table 2:** Baseline characteristics of UK Biobank participants.

Characteristic	Total (*n* = 308,156)	Women (*n* = 163,022)	Men (*n* = 145,134)	*P*^a^
Chronological age at baseline (year), mean ± SD	56.21 ± 8.11	56.09 ± 8.01	56.35 ± 8.21	<0.001
BA measures^b^, mean ± SD				
KDM (year)	53.94 ± 9.42	53.90 ± 9.53	53.99 ± 9.29	0.006
KDM residual	−0.04 ± 5.01	0.03 ± 5.03	−0.12 ± 4.98	<0.001
PhenoAge (year)	47.46 ± 10.00	46.70 ± 9.52	48.31 ± 10.46	<0.001
PhenoAge residual	−0.03 ± 5.35	−0.65 ± 5.17	0.67 ± 5.46	<0.001
HD (log units)	6.69 ± 1.00	6.81 ± 0.99	6.54 ± 1.00	<0.001
Biomarkers included in BA algorithms, mean ± SD				
FEV_1_ (L)	2.84 ± 0.80	2.41 ± 0.55	3.32 ± 0.77	<0.001
SBP (mm Hg)	139.55 ± 19.50	136.88 ± 20.12	142.56 ± 18.33	<0.001
Blood urea nitrogen (mg/dL)	15.08 ± 3.76	14.58 ± 3.62	15.64 ± 3.84	<0.001
HbA1c (%)	5.44 ± 0.60	5.41 ± 0.53	5.47 ± 0.66	<0.001
Total cholesterol (mg/dL)	220.48 ± 43.87	227.17 ± 43.23	212.97 ± 43.36	<0.001
Creatinine (umol/L)	72.29 ± 15.23	64.18 ± 11.00	81.40 ± 14.13	<0.001
Serum glucose (mmol/L)	5.11 ± 1.19	5.05 ± 1.02	5.17 ± 1.36	<0.001
Waist circumference (cm)	90.23 ± 13.34	84.42 ± 12.38	96.76 ± 11.19	<0.001
Red cell distribution width (%)	13.46 ± 0.94	13.51 ± 1.03	13.42 ± 0.82	<0.001
Albumin (g/dL)	45.30 ± 2.59	45.01 ± 2.57	45.62 ± 2.58	<0.001
Alkaline phosphatase (U/L)	82.88 ± 24.80	84.07 ± 25.90	81.54 ± 23.44	<0.001
Triglyceride (mg/dL)	153.81 ± 89.83	135.51 ± 74.31	174.37 ± 100.65	<0.001
Mean cell volume (fL)	82.77 ± 5.28	82.89 ± 5.28	82.63 ± 5.27	<0.001
Uric acid (mg/dL)	5.19 ± 1.34	4.52 ± 1.09	5.95 ± 1.19	<0.001
Lymphocyte (%)	29.03 ± 7.34	29.89 ± 7.26	28.06 ± 7.31	<0.001
RBC count (million cells/uL)	4.53 ± 0.41	4.33 ± 0.33	4.76 ± 0.37	<0.001
C-reactive protein (mg/dL)	0.25 ± 0.40	0.26 ± 0.41	0.23 ± 0.40	<0.001
DBP (mm Hg)	82.24 ± 10.64	80.61 ± 10.51	84.07 ± 10.49	<0.001
Died during follow-up, *n* (%)	14,542 (4.7)	5495 (3.4)	9047 (6.2)	<0.001
Any incident cancer^c^, *n* (%)	35,426 (11.5)	16,933 (10.4)	18,493 (12.7)	<0.001
Incident breast cancer in women, *n* (%)	—	5794 (3.6)	—	—
Incident prostate cancer in men, *n* (%)	—	—	7196 (5.0)	—
Incident lung cancer, *n* (%)	2363 (0.8)	1109 (0.7)	1254 (0.9)	<0.001
Incident colorectal cancer, *n* (%)	3596 (1.2)	1552 (1.0)	2044 (1.4)	<0.001
Incident melanoma, *n* (%)	1833 (0.6)	881 (0.5)	952 (0.7)	<0.001

*BA* biological age, *DBP* diastolic blood pressure, *FEV*_*1*_ forced expiratory volume in 1 second, *HbA1c* glycated hemoglobin, *HD* homeostatic dysregulation, *KDM* Klemera-Doubal method, *RBC* red blood cell, *SBP* systolic blood pressure, *SD* standard deviation.

^a^*P* values for comparison between women and men, obtained from *t* tests or *χ*^2^ tests.

^b^All algorithms were parametrized in NHANES III and projected in UK Biobank. The KDM residual and PhenoAge residual were computed by regressing out chronological age (as a natural spline term with 3 degrees of freedom) from the KDM and PhenoAge, respectively.

^c^Any type of cancer, except non-melanoma skin cancer.

**Fig. 1:**
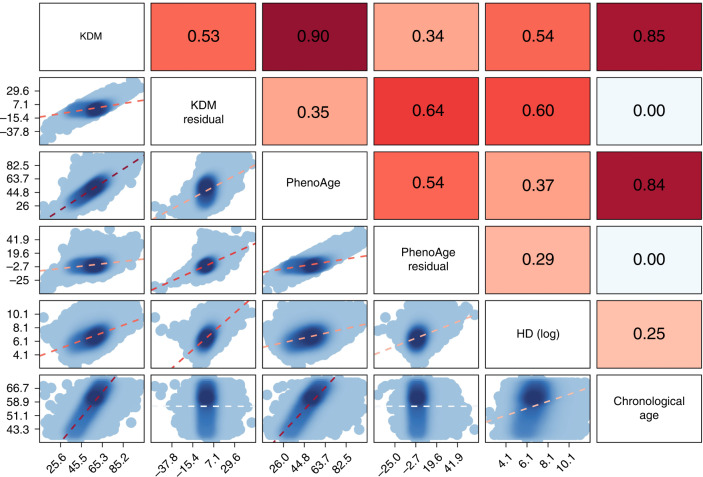
Correlations between biological age measures and chronological age in UK Biobank (*n* = 308,156). The KDM residual and PhenoAge residual were computed by regressing out chronological age (as a natural spline term with three degrees of freedom) from the KDM-biological age and PhenoAge, respectively. HD homeostatic dysregulation, KDM Klemera-Doubal method.

Correlations between the BA measures and CA in UK Biobank are shown in Fig. 1. As expected, CA was strongly correlated with KDM (i.e., an algorithm representing predicted CA; *r* = 0.85) and PhenoAge (i.e., representing predicted age-associated mortality; *r* = 0.84), while a weaker correlation was found between CA and HD (i.e., representing deviation of physiology from a healthy reference; *r* = 0.25). The residual (i.e., removing the effect of CA) of KDM was moderately correlated with the PhenoAge residual (*r* = 0.64) and HD (*r* = 0.60), but there was only weak correlation between the PhenoAge residual and HD (*r* = 0.29). Compared to men, women had a higher mean KDM residual (0.03 vs. −0.12) and HD (6.81 vs. 6.54 log units), but a lower mean PhenoAge residual (−0.65 vs. 0.67) (Table 2).

### Biological age and cancer incidence

After adjusting for sociodemographic and lifestyle factors, all BA measures were associated with an elevated risk of any cancer (KDM residual: HR per 1-SD increase=1.04, 95% confidence interval [CI] = 1.03–1.05; PhenoAge residual: 1.09, 1.07–1.10; HD: 1.02, 1.01–1.03) (Table 3 and Supplementary Fig. 5). Similarly, the BA measures were associated with increased risks of lung cancer and colorectal cancer in the full models adjusted for cancer-specific risk factors. Among the three measures, PhenoAge residual had the strongest effect estimate for lung cancer (HR = 1.35, 95% CI = 1.30–1.40), and HD had the strongest effect estimate for colorectal cancer (HR = 1.10, 95% CI = 1.07–1.15). Only PhenoAge residual (HR = 1.05, 95% CI = 1.02–1.08), but not KDM residual or HD, was associated with a higher risk of breast cancer. We also observed significant protective effects of KDM and PhenoAge residuals for prostate cancer, although the associations were slightly attenuated after adjusting for prostate cancer-specific factors. None of the BA measures were statistically significantly associated with melanoma after adjustment for sociodemographic and lifestyle factors (Table 3). Many of the 18 clinical biomarkers incorporated within BA measures were also associated with cancer risks individually (Supplementary Table 4 and Supplementary Fig. 5). For instance, higher FEV_1_ was associated with decreased risks of any cancer and lung cancer but increased risk of prostate cancer, and higher systolic blood pressure was associated with elevated risk of breast cancer.

**Table 3:** Association between biological age measures and risk of cancer in UK Biobank.

Cancer site	KDM residual		PhenoAge residual		HD (log)	
	HR per 1 SD increase (95% CI)	*P*	HR per 1 SD increase (95% CI)	*P*	HR per 1 SD increase (95% CI)	*P*
Any cancer						
Age- and sex-adjusted model^a^	1.06 (1.05, 1.07)*	<0.001	1.10 (1.09, 1.11)*	<0.001	1.04 (1.03, 1.05)*	<0.001
Multivariable model^b^	1.04 (1.03, 1.05)*	<0.001	1.09 (1.07, 1.10)*	<0.001	1.02 (1.01, 1.03)*	0.002
Breast cancer in women						
Age-adjusted model^a^	1.02 (1.00, 1.05)	0.09	1.05 (1.03, 1.08)*	<0.001	1.02 (0.99, 1.05)	0.26
Multivariable model^b^	1.01 (0.98, 1.04)	0.69	1.06 (1.03, 1.09)*	<0.001	1.00 (0.97, 1.03)	0.99
Breast cancer-specific model^c^	1.01 (0.98, 1.04)	0.51	1.05 (1.02, 1.08)*	<0.001	1.00 (0.97, 1.03)	0.86
Prostate cancer in men						
Age-adjusted model^a^	0.92 (0.90, 0.94)*	<0.001	0.94 (0.91, 0.96)*	<0.001	0.93 (0.91, 0.96)*	<0.001
Multivariable model^b^	0.94 (0.92, 0.97)*	<0.001	0.95 (0.93, 0.97)*	<0.001	0.96 (0.93, 0.98)*	<0.001
Prostate cancer-specific model^d^	0.96 (0.93, 0.98)*	0.002	0.96 (0.94, 0.99)*	0.003	0.97 (0.95, 1.00)	0.044
Lung cancer						
Age- and sex-adjusted model^a^	1.44 (1.39, 1.50)*	<0.001	1.58 (1.53, 1.63)*	<0.001	1.22 (1.17, 1.27)*	<0.001
Multivariable model^b^	1.29 (1.24, 1.35)*	<0.001	1.35 (1.30, 1.40)*	<0.001	1.12 (1.08, 1.18)*	<0.001
Lung cancer-specific model^e^	1.29 (1.24, 1.35)*	<0.001	1.35 (1.30, 1.40)*	<0.001	1.13 (1.08, 1.18)*	<0.001
Colorectal cancer						
Age- and sex-adjusted model^a^	1.10 (1.07, 1.14)*	<0.001	1.09 (1.06, 1.13)*	<0.001	1.13 (1.09, 1.17)*	<0.001
Multivariable model^b^	1.09 (1.05, 1.13)*	<0.001	1.09 (1.05, 1.12)*	<0.001	1.11 (1.07, 1.15)*	<0.001
Colorectal cancer-specific model^f^	1.09 (1.05, 1.12)*	<0.001	1.08 (1.05, 1.12)*	<0.001	1.10 (1.07, 1.15)*	<0.001
Melanoma						
Age- and sex-adjusted model^a^	0.91 (0.86, 0.95)*	<0.001	0.94 (0.89, 0.98)	0.008	0.93 (0.89, 0.98)	0.006
Multivariable model^b^	0.95 (0.90, 1.00)	0.040	1.01 (0.96, 1.06)	0.85	0.95 (0.91, 1.01)	0.08
Melanoma-specific model^g^	0.95 (0.90, 1.00)	0.062	1.01 (0.96, 1.07)	0.62	0.95 (0.91, 1.01)	0.09

*CI* confidence interval, *HD* homeostatic dysregulation, *HR* hazard ratio, *KDM* Klemera-Doubal method, *SD* standard deviation.

^a^Age- and sex-adjusted model: adjusted for age (time scale), birth year (1930–1939, 1940–1949, 1950–1959, ≥1960), and sex (except for breast cancer and prostate cancer).

^b^Multivariable model: age- and sex-adjusted model + baseline assessment center (England, Wales, Scotland), ethnic background (White, Asian, Black, others), body mass index (underweight, normal weight, overweight, obese), smoking status (never, previous, current), physical activity level (low, moderate, high), alcohol consumption (less than 3 times a month, 1–4 times a week, daily or almost daily), education level (high, intermediate, low), deprivation index quintiles (1st, 2nd, 3rd, 4th, 5th).

^c^Breast cancer-specific model: multivariable model + family history of breast cancer (no, yes), ever had breast cancer screening (no, yes), menopause (premenopausal, postmenopausal), hormone replacement therapy use (never, ever), oral contraceptive use (never, ever), parity (0, 1–2, ≥3).

^d^Prostate cancer-specific model: multivariable model + family history of prostate cancer (no, yes), ever had prostate-specific antigen test (no, yes), self-reported diabetes (no, yes).

^e^Lung cancer-specific model: multivariable model + family history of lung cancer (no, yes).

^f^Colorectal cancer-specific model: multivariable model + family history of colorectal cancer (no, yes), ever had colorectal cancer screening (no, yes), fresh vegetable and fruit intake (<5 portions a day, ≥5 portions a day), red meat intake (less than twice a week, twice a week or more), processed meat intake (less than twice a week, twice a week or more).

^g^Melanoma cancer-specific model: multivariable model + time spent outdoors during summer (1–2 h/day, 3–5 h/day, >5 h/day), use of sun/UV protection (never/rarely, sometimes, most of the time, always, do not go out in sunshine), sunburn during childhood (no, yes), solarium/sunlamp use (no, yes), ease of skin tanning (very tanned, moderately tanned, mildly or occasionally tanned, never tan but only burn), skin color (black/brown, light/dark olive, fair, very fair), hair color (black/dark brown/other, light brown, blonde/red).

*Significant after Bonferroni correction at *P* < 0.05/15 (i.e., 5 cancers × 3 biological age measures).

Associations between BA measures and cancer risks were mostly consistent across subgroups split by age and sex; however, the associations of KDM and PhenoAge residuals with colorectal cancer appeared to be sexually dimorphic and were only significant among men (Supplementary Table 5). When stratifying by ethnicity, results in non-white participants were generally statistically non-significant, possibly due to the limited sample size. Moreover, the associations between BA measures and lung cancer were only significant among ever-smokers but not never-smokers (*P* for interaction < 0.001), and the association between PhenoAge and breast cancer was stronger in postmenopausal vs. premenopausal women (*P* for interaction = 0.002) (Supplementary Table 5).

**Fig. 2:**
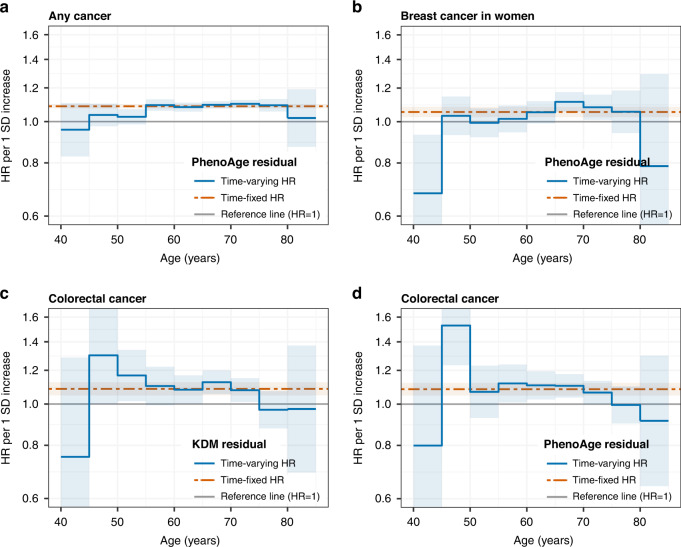
Time-varying hazard ratios for risk of cancers per 1 standard deviation increase in biological age measures in UK Biobank (*n* = 308,156). **a** PhenoAge residual and risk of any cancer; **b** PhenoAge residual and risk of breast cancer in women; **c** KDM residual and risk of colorectal cancer; **d** PhenoAge residual and risk of colorectal cancer. Only the models with evidence of non-proportional hazards (*P* < 0.05) are shown. Estimates were obtained by including interaction terms between the exposure and the time variable (i.e., attained age, split into 5-year intervals) in the Cox models. The shaded areas indicate 95% confidence intervals (HRs outside the boundaries between 0.6 and 1.6 are not shown). The proportional hazards assumption of the biological age measures fitted in the Cox models was tested using Schoenfeld residuals. All models were adjusted for age (time scale), birth year, sex, baseline assessment center, ethnic background, body mass index, smoking status, physical activity level, alcohol consumption, education level, deprivation index quintiles, and the cancer-specific covariates as detailed in the footnote of Table 3 and in Supplementary Table 3. HR hazard ratio, KDM Klemera-Doubal method, SD standard deviation.

**Fig. 3:**
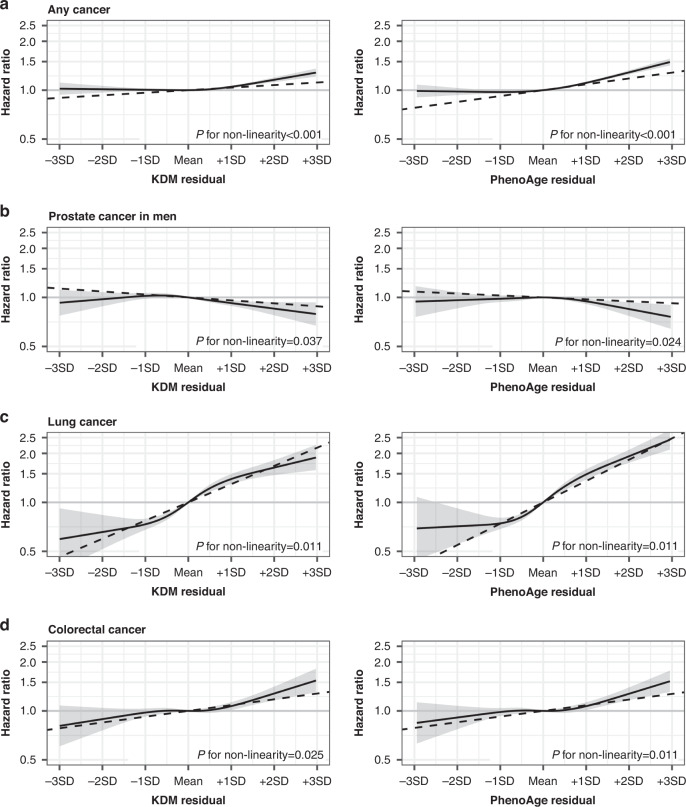
Dose-response relationships between biological age measures and risk of cancer in UK Biobank (*n* = 308,156). **a** Any cancer; **b** Prostate cancer in men; **c** Lung cancer; **d** Colorectal cancer. Only the models with evidence of non-linearity (*P* < 0.05) are shown. The black solid lines represent hazard ratios and the corresponding 95% confidence intervals (shaded areas) estimated using restricted cubic spline Cox regression models with knots at the 25th, 50th, and 75th percentiles. The dashed lines represent the estimates obtained from models assuming linear relationships. The mean of each BA measure was used as the reference value. *P* values for non-linearity were from likelihood ratio tests comparing the spline models with linear models. All models were adjusted for age (time scale), birth year, sex, baseline assessment center, ethnic background, body mass index, smoking status, physical activity level, alcohol consumption, education level, deprivation index quintiles, and the cancer-specific covariates as detailed in the footnote of Table 3 and in Supplementary Table 3. HD homeostatic dysregulation, KDM Klemera-Doubal method, SD standard deviation.

We calculated age-varying HRs when the proportional-hazards assumption was not met. As shown in Fig. 2, the associations of PhenoAge residual with any cancer and breast cancer appeared to be stronger around 60–80 years, while the associations of PhenoAge and KDM residuals with colorectal cancer tended to be weaker with advancing age. There was some evidence of non-linear associations between the residuals of KDM and PhenoAge and the risk of any cancer, lung cancer, and colorectal cancer (*P* < 0.05), with a sharp increase in the risk of any cancer among individuals with a BA value above the mean (Fig. 3).

As the individual biomarkers included in the BA measures had different effects on cancer risks (Supplementary Fig. 5), we performed sensitivity analysis using alternative BA algorithms incorporating other sets of biomarkers. Results were essentially unchanged when using the original KDM and PhenoAge algorithms (Supplementary Table 6), as well as the modified algorithms of KDM, PhenoAge, and HD (excluding HbA1c and serum glucose) as exposure variables (Supplementary Table 7), although these alternative algorithms were no longer statistically significantly associated with prostate cancer in the fully-adjusted models. The associations were also largely similar when performing the complete-case analysis rather than using indicator variables for missing values of covariates (Supplementary Table 8), and when excluding diagnoses occurring in the first 2 years of follow-up (Supplementary Table 9).

## Discussion

In this large cohort of UK Biobank participants, we quantified BA based on clinical biomarkers (e.g., blood chemistries, blood pressure, lung function) and showed that they were associated with increased risks of any cancer, lung cancer, and colorectal cancer, independent of CA, sex, sociodemographic and lifestyle factors, and other cancer-specific risk factors. These associations were largely consistent over the follow-up period and across subgroups split by age and sex. We also found a positive association between the age-adjusted PhenoAge and breast cancer. Furthermore, a protective effect of an advanced BA on prostate cancer was seen in the main analysis, although this seemed to be primarily driven by HbA1c and serum glucose included in the BA algorithms.

There is no gold standard for measuring BA. Different BA measures (e.g., telomere length, epigenetic clocks, biomarker-based BA) are weakly correlated with each other, hence, they may explain different aspects of biological aging [19, 20]. To the best of our knowledge, this is the first study that has used clinical biomarker-based BA algorithms to predict cancer risks. Similar to previous studies [14–18, 32, 33], we found that individual clinical biomarkers had different effects on various cancer types. Importantly, when combining information from multiple biomarkers as the proxies for biological aging (i.e., reflecting the overall physiological status of a person), these composite measures of BA had modest, but significant associations with any cancer, lung cancer, and colorectal cancer. Our results are also consistent with the existing literature, which has shown an association between higher epigenetic ages and an increased risk of any cancer [9–11], lung cancer [11, 34, 35], and colorectal cancer [13, 34]. Taken together, these findings may indicate that multiple aging processes captured by BA measures (e.g., epigenetic alterations, inflammation, metabolic changes) could play a role in cancer development. Although our BA algorithms included FEV_1_, which is a lung function biomarker strongly associated with lung cancer [18], we also observed a strong association between the original Levine PhenoAge algorithm (without FEV_1_ included) and lung cancer, thus suggesting that the association between BA measures and lung cancer was not entirely due to reduced lung function. Meanwhile, the association between BA measures and lung cancer was only significant among ever-smokers, but not never-smokers, indicating that it could partly be confounded by smoking status, where smoking could lead to both advanced BA and lung cancer risk [36]. Further research is warranted to study the mechanisms underlying biological aging, lung cancer and colorectal cancer.

We studied three algorithms, the KDM, PhenoAge, and HD, which were trained using different methods and may therefore have slightly different implications on biological aging. While the associations of the three BA measures and most cancers were comparable, we only observed a statistically significant association of PhenoAge, but not KDM or HD, with breast cancer. This is similar to prior research showing a statistically significant association between the PhenoAge clock (i.e., a DNA methylation-based algorithm trained to predict the PhenoAge used in our study) and increased risk of breast cancer [37], but a weaker association when using Horvath and Hannum clocks (i.e., algorithms to predict CA) [37, 38]. As we incorporated the same set of biomarkers into our three BA measures, the inconsistent result is probably explained by the fact that PhenoAge captures not merely CA, but also the mortality risk predicted by the biomarkers, whereas KDM simply reflects CA and HD reflects physiological deviations from a healthy reference.

The inverse association between BA and prostate cancer found in our primary analysis is somewhat in line with previous studies which showed indications of a protective effect of the GrimAge clock for prostate cancer [13, 34]. Interestingly, in our study, this association was slightly attenuated after adjusting for prostate cancer-specific factors such as diabetes and having prostate-specific antigen (PSA) test, and was further attenuated to non-statistically significance after removing HbA1c and serum glucose from the BA algorithms. Diabetes [39], as well as HbA1c [32] and serum glucose levels [33], have been linked to a lower incidence of prostate cancer, partly because of the lower level of insulin-like growth factor-1 levels in diabetic patients, and partly because of the potential detection bias due to the lower PSA level in diabetic men [32, 39]. We therefore speculate that the apparent protective effect of higher BA for prostate cancer could be confounded by diabetes and altered glucose metabolism, which are also closely related to aging [40]. Finally, no significant association was found between BA measures and melanoma. As melanoma is a cancer that is relatively common in young adults [30], it may be less driven by systemic aging and more by other factors such as skin sun exposure.

The primary strength of this study is the large sample size and the relatively long follow-up of ~10 years, which have allowed us to assess several cancers. Using the *BioAge* R package [25], we were able to assess the impact of multiple BA algorithms and compositions on cancer risks. Nevertheless, one limitation is the lack of data on tumor stage and grade. We therefore could not account for the severity of cancers. As we only have information on the clinical biomarkers at baseline, we were also unable to analyze if changes in BA over time may influence cancer risks. Moreover, the UK Biobank sample was mostly white participants (>94%), which may limit the generalizability of our results to other populations. Finally, as in other observational studies, although we have carefully adjusted for several cancer risk factors, the possibility of residual and unmeasured confounding cannot be ruled out.

In conclusion, our findings suggest that advanced biological aging may lead to increased risks of any cancer, lung cancer, and colorectal cancer, independent of age, sex, and common cancer risk factors. However, for other cancers such as breast cancer and prostate cancer, the associations may be influenced by the algorithm and composition of the BA being used. This work provides the basis for our further understanding on the biology underlying aging and cancer, and suggests that slowing down biological aging may be beneficial to mitigate cancer risks.

## Supplementary information

Supplementary Figures and Tables

## Data Availability

UK Biobank is an open access resource. All bona fide researchers can apply to use its data for health-related research that is in the public interest (http://www.ukbiobank.ac.uk/register-apply).

## Code availability

All codes used for generating the biological age measures and for conducting statistical analyses are available at https://github.com/jonathanklmak/UKB_bioage_cancer.
