# Supplementary material for: Clinical biomarker-based biological aging and risk of cancer in the UK Biobank
Source: Br J Cancer. 2023 Apr 29;129(1):94–103. doi: 10.1038/s41416-023-02288-w (PMC10307789; doi:10.1038/s41416-023-02288-w)
Supplement: Supplementary file 1 — Supplementary Figures and Tables [file 41416_2023_2288_MOESM1_ESM.pdf]

Clinical biomarker-based biological aging and risk of cancer in the UK Biobank

Jonathan K. L. Mak[1], Christopher E. McMurran[1,2], Ralf Kuja-Halkola[1], Per Hall[1,3], Kamila Czene[1], Juulia Jylhävä[1,4], Sara Hägg[1]

1 Department of Medical Epidemiology and Biostatistics, Karolinska Institutet, Stockholm, Sweden
2 Department of Clinical Neurosciences, University of Cambridge, Cambridge, United Kingdom
3 Department of Oncology, Södersjukhuset, Stockholm, Sweden
4 Faculty of Social Sciences (Health Sciences) and Gerontology Research Center (GEREC), University of Tampere, Tampere, Finland

Correspondence: Jonathan K. L. Mak (jonathan.mak@ki.se)

Supplementary Material

Supplementary Figure 1. Density plots of biomarkers in individuals aged 37–73 years in NHANES III (white) and UK Biobank (pink)........ 2

Supplementary Figure 2. Scatter plots of biological age measures and chronological age in NHANES IV (n=3,851) 3

Supplementary Figure 3. Correlations among biological age measures and chronological age in NHANES IV (n=3,851) 4

Supplementary Figure 4. Density plots of biological age measures in UK Biobank (n=308,156) 5

Supplementary Figure 5. Summary of the associations of clinical biomarkers and biological age measures with cancer outcomes in UK Biobank (n=308,156) 6

Supplementary Table 1. Associations of biological age measures with mortality in NHANES IV........ 7

Supplementary Table 2. Correlations among the biomarkers used for construction of the biological age measures in the UK Biobank (n=308,156) 8

Supplementary Table 3. Descriptive statistics for covariates in UK Biobank. Data are shown as numbers (%). ... 9

Supplementary Table 4. Associations between individual clinical biomarkers and risk of cancer in UK Biobank (n=308,156) 12

Supplementary Table 5. Subgroup analyses for the associations between biological age measures and risk of cancer in UK Biobank........ 13

Supplementary Table 6. Associations between biological age measures using the Levine original KDM and PhenoAge algorithms and risk of cancer in UK Biobank 15

Supplementary Table 7. Associations between modified biological age measures excluding HbA1c and serum glucose from the algorithms and risk of cancer in UK Biobank........ 16

Supplementary Table 8. Associations between biological age measures and risk of cancer in using complete data in UK Biobank........ 17

Supplementary Table 9. Associations between biological age measures and risk of cancer excluding individuals with <2 years follow-up........ 18

Supplementary Figure 1. Density plots of biomarkers in individuals aged 37–73 years in NHANES III (white) and UK Biobank (pink)

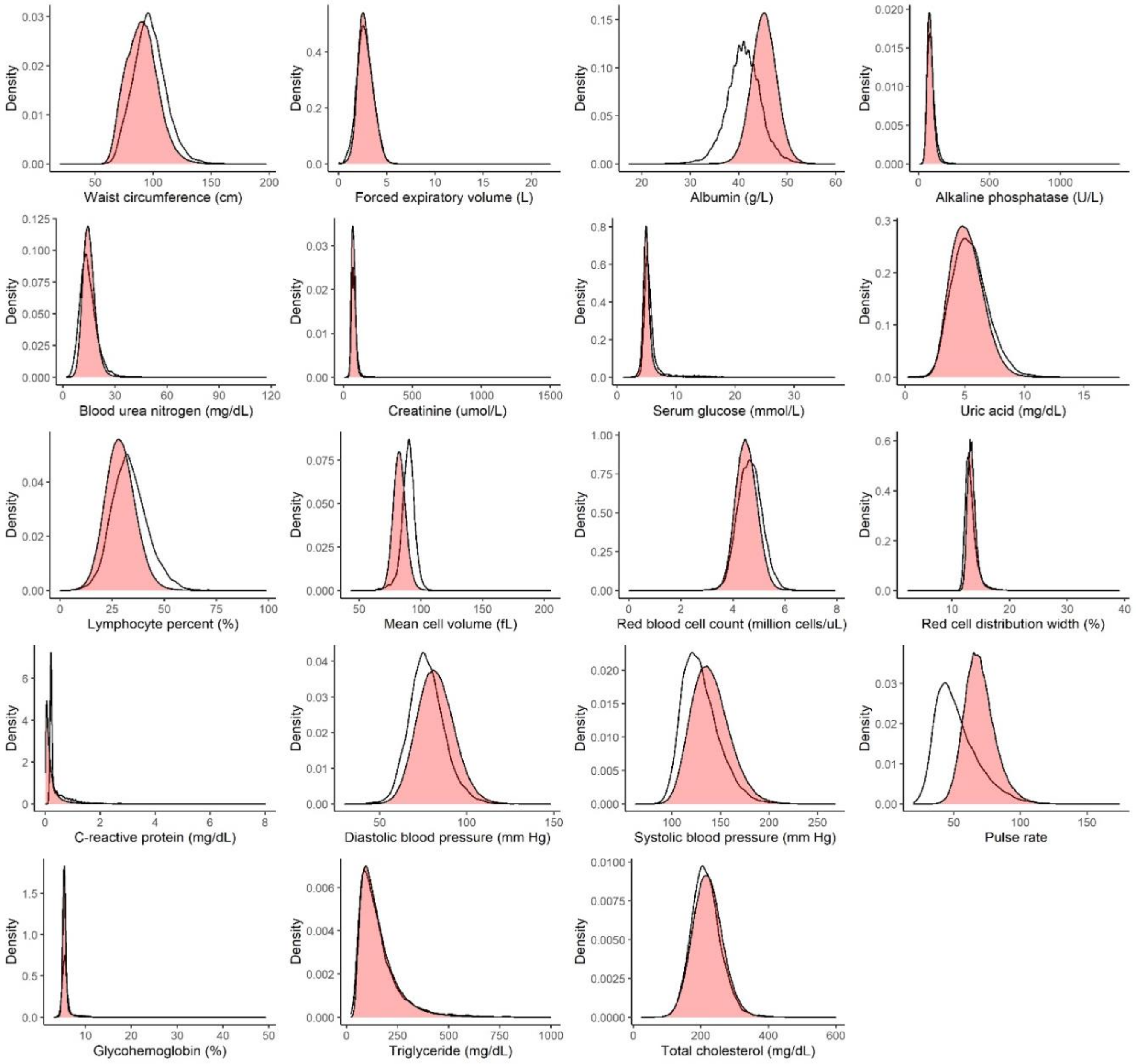

Pulse was not included in the final biological age algorithms it had high correlation with systolic blood pressure (*r*=.84) (i.e., the models did not converge when including it). Also, the distribution of pulse in NHANES III and UKB was slightly different as shown in this figure. *Abbreviations*: *NHANES*, National Health and Nutrition Examination Survey.

Supplementary Figure 2. Scatter plots of biological age measures and chronological age in NHANES IV (n=3,851)

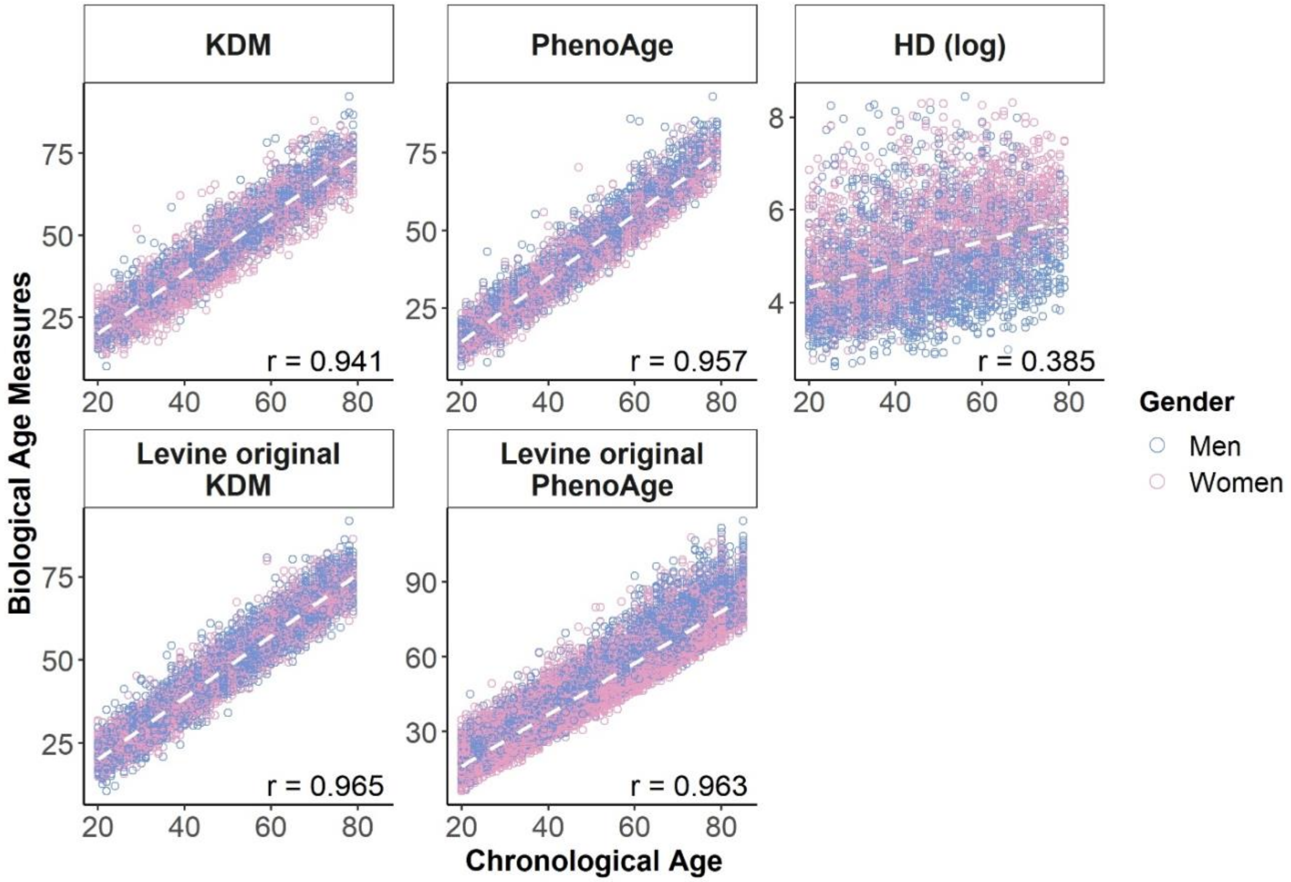

Abbreviations: *HD*, homeostatic dysregulation; *KDM*, Klemera-Doubal method; *NHANES*, National Health and Nutrition Examination Survey

Supplementary Figure 3. Correlations among biological age measures and chronological age in NHANES IV (n=3,851)

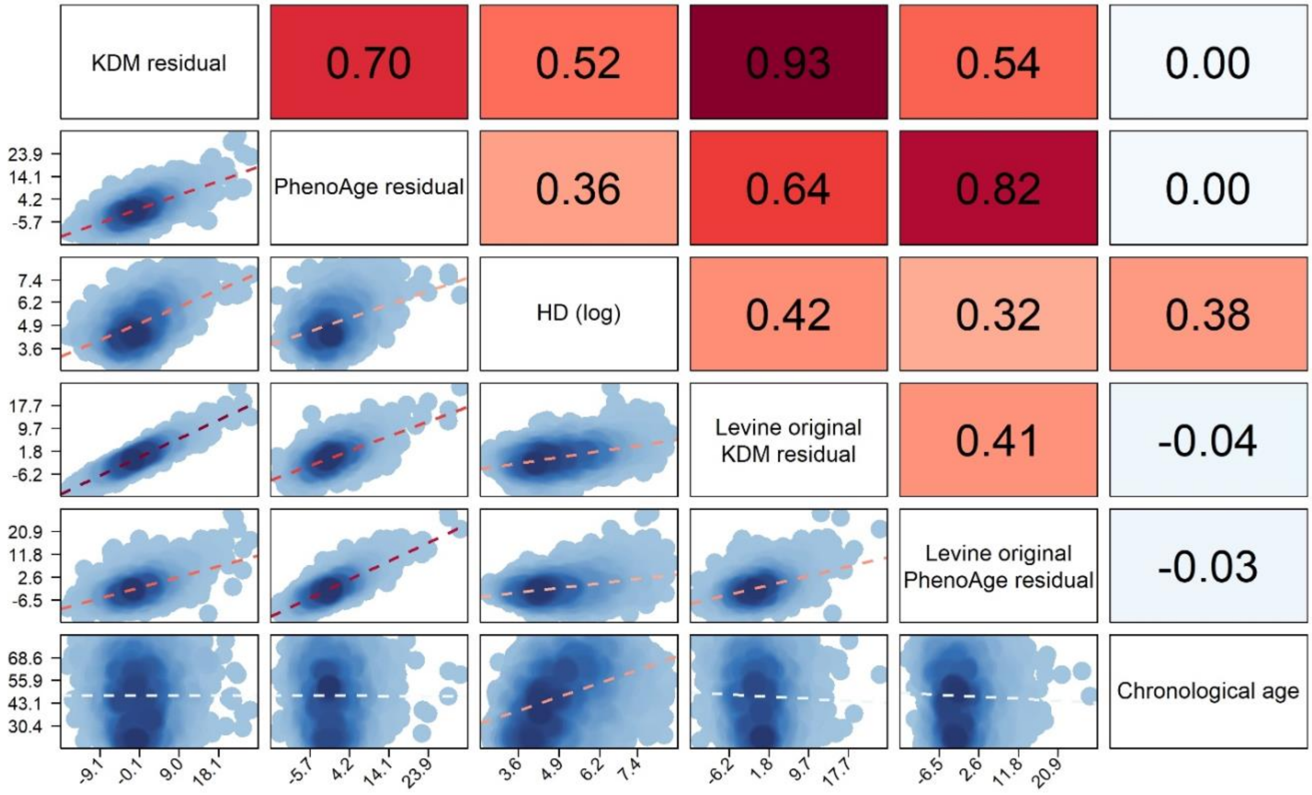

The KDM residual and PhenoAge residual were computed by regressing out chronological age (as a natural spline term with three degrees of freedom) from the KDM-biological age and PhenoAge, respectively. *Abbreviations*: *HD*, homeostatic dysregulation; *KDM*, Klemera-Doubal method; *NHANES*, National Health and Nutrition Examination Survey

Supplementary Figure 4. Density plots of biological age measures in UK Biobank (n=308,156)

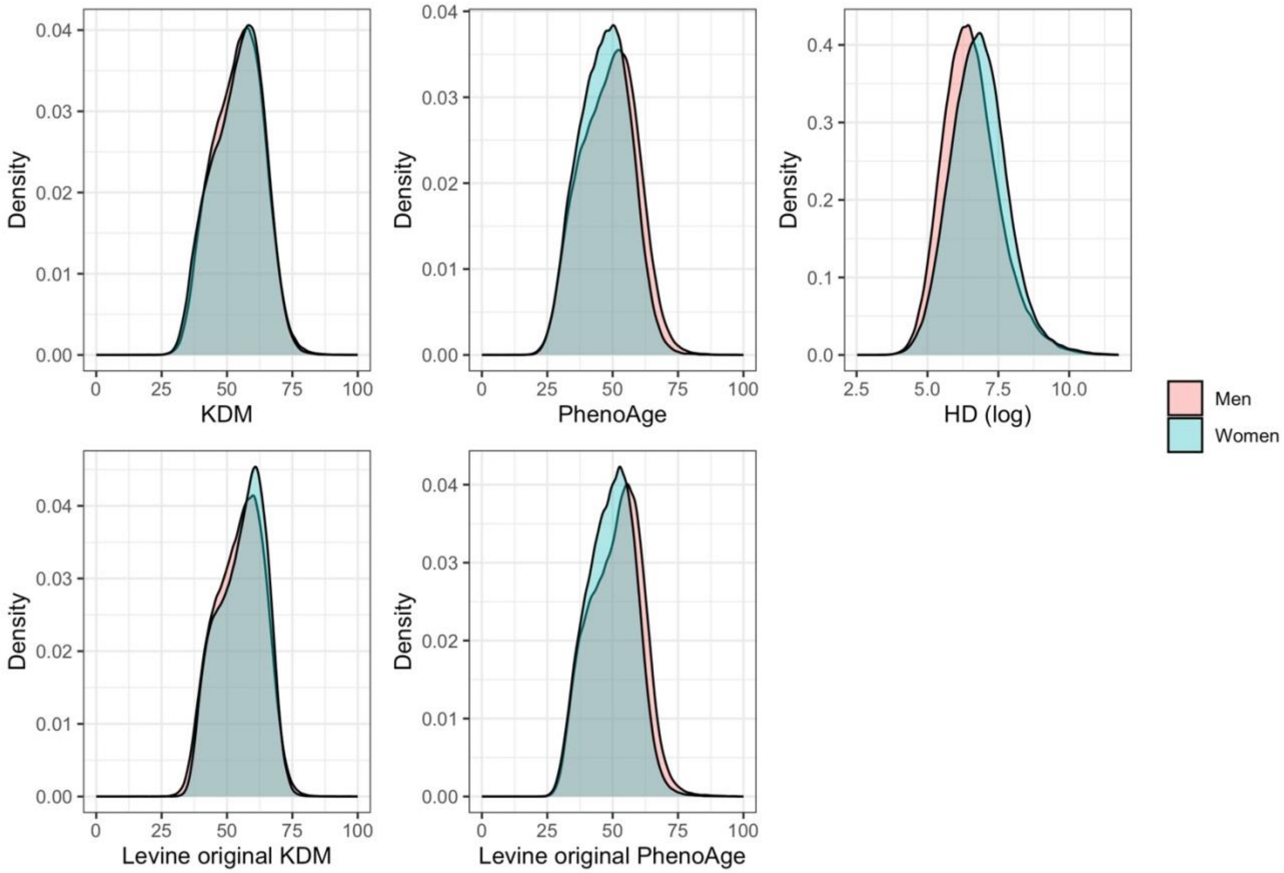

Abbreviations: *HD*, homeostatic dysregulation; *KDM*, Klemera-Doubal method

Supplementary Figure 5. Summary of the associations of clinical biomarkers and biological age measures with cancer outcomes in UK Biobank (n=308,156)

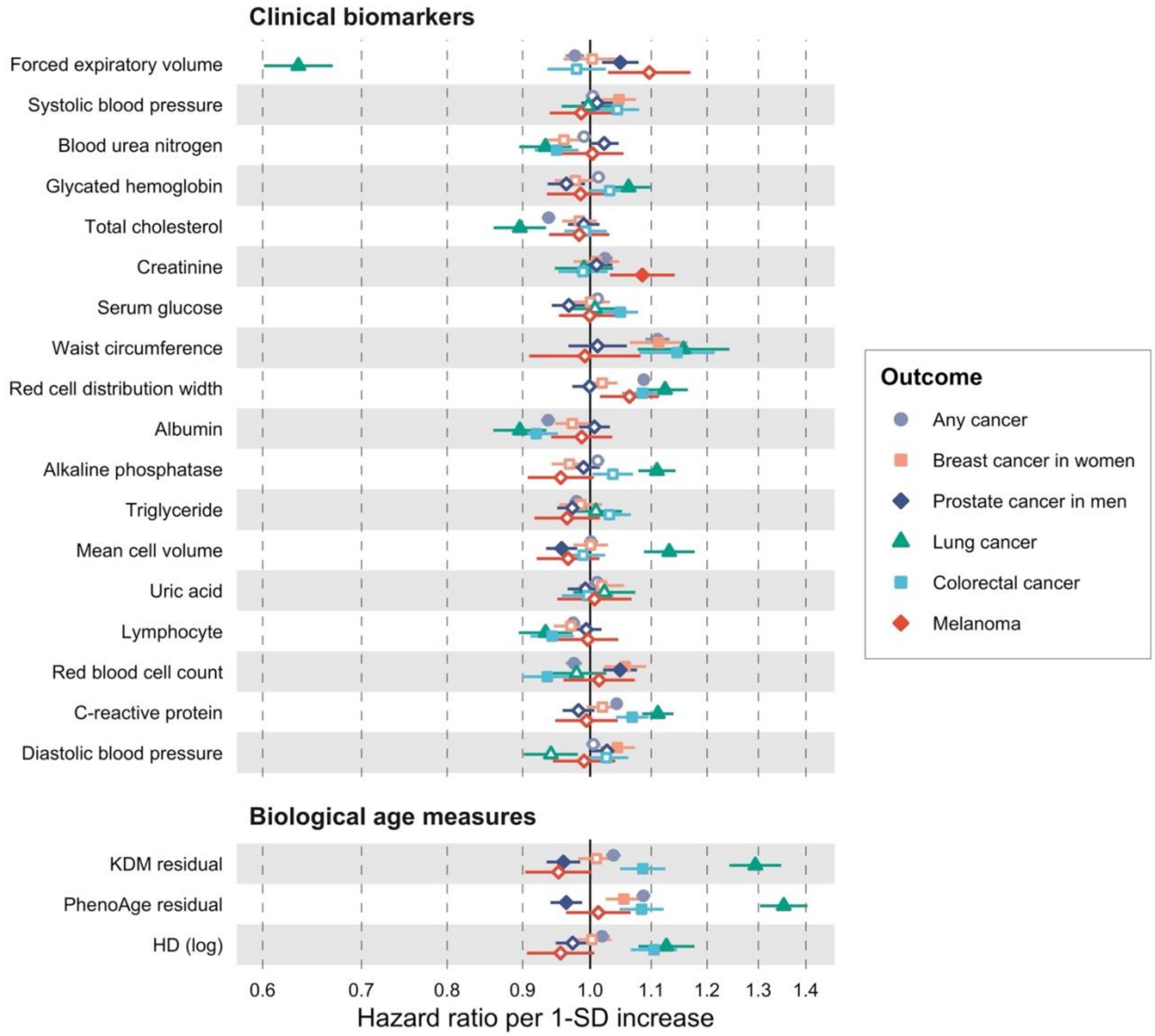

Filled symbols represent significant associations after Bonferroni correction at *p*<.05/15 (i.e., 5 cancers × 3 biological age measures). All models were adjusted for age (time scale), birth year, sex, baseline assessment center, ethnic background, body mass index, smoking status, physical activity level, alcohol consumption, education level, deprivation index quintiles, and the cancer-specific covariates as detailed in the Supplementary Table 3 and the footnote of Table 3. All estimates are represented in Table 3 and Supplementary Table 4. *Abbreviations*: *HD*, homeostatic dysregulation; *KDM*, Klemera-Doubal method; *SD*, standard deviation

Supplementary Table 1. Associations of biological age measures with mortality in NHANES IV

	KDM residual	PhenoAge residual	HD (log)	Levine original KDM residual	Levine original PhenoAge residual
**Full sample**					
n	3851	3851	3851	8234	27837
HR (95% CI)	1.66 (1.39, 1.97)	1.84 (1.60, 2.13)	1.55 (1.24, 1.94)	1.35 (1.19, 1.53)	1.48 (1.44, 1.53)
**Stratified by sex**					
Men					
n	1921	1921	1921	4114	13421
HR (95% CI)	1.84 (1.50, 2.26)	1.82 (1.54, 2.16)	1.61 (1.23, 2.09)	1.42 (1.21, 1.65)	1.44 (1.38, 1.50)
Women					
n	1930	1930	1930	4120	14416
HR (95% CI)	1.34 (0.97, 1.84)	1.97 (1.49, 2.61)	1.43 (0.95, 2.17)	1.22 (0.99, 1.52)	1.57 (1.49, 1.65)
**Stratified by race**					
White					
n	1811	1811	1811	3937	13958
HR (95% CI)	1.63 (1.29, 2.05)	2.04 (1.62, 2.57)	1.55 (1.16, 2.06)	1.42 (1.20, 1.68)	1.54 (1.48, 1.61)
Black					
n	675	675	675	1467	5176
HR (95% CI)	1.92 (1.36, 2.71)	1.65 (1.24, 2.20)	2.83 (1.66, 4.81)	1.48 (1.14, 1.93)	1.39 (1.30, 1.48)
Other					
n	1365	1365	1365	2830	8703
HR (95% CI)	1.74 (1.09, 2.79)	2.54 (1.53, 4.23)	0.93 (0.56, 1.67)	1.19 (0.89, 1.61)	1.40 (1.29, 1.51)
**Aged 65 or younger**					
n	3244	3244	3244	6915	21252
HR (95% CI)	1.59 (1.26, 2.02)	1.75 (1.47, 2.09)	1.41 (1.06, 1.86)	1.26 (1.06, 1.51)	1.67 (1.57, 1.77)

Abbreviations: *HD*, homeostatic dysregulation; *HR*, hazard ratio; *KDM*, Klemera-Doubal method; *NHANES*, National Health and Nutrition Examination Survey. Hazard ratios are per 1 standard deviation increase in the biological age measure. Estimates were obtained from Cox proportional-hazards models, adjusted for chronological age and sex. The “Levine original KDM” and “Levine original PhenoAge” were calculated using the original list of biomarkers included in Levine 2013 and Levine et al. 2018, as shown in Table 1.

Supplementary Table 2. Correlations among the biomarkers used for construction of the biological age measures in the UK Biobank (n=308,156)

#	Biomarker	Pearson's correlation between biomarkers																	
		**1**	**2**	**3**	**4**	**5**	**6**	**7**	**8**	**9**	**10**	**11**	**12**	**13**	**14**	**15**	**16**	**17**	**18**
**1**	FEV$_1$ (L)	1																	
**2**	SBP (mm Hg)	-0.07	1																
**3**	Blood urea nitrogen (mg/dL)	0.01	0.07	1															
**4**	HbA1c (%)	-0.15	0.10	0.10	1														
**5**	Total cholesterol (mg/dL)	-0.08	0.10	-0.01	-0.09	1													
**6**	Creatinine (µmol/L)	0.32	0.08	0.44	0.03	-0.13	1												
**7**	Serum glucose (mmol/L)	-0.06	0.12	0.06	0.62	-0.09	0.00	1											
**8**	Waist circumference (cm)	0.16	0.22	0.14	0.25	-0.09	0.31	0.17	1										
**9**	Red cell distribution width (%)	-0.10	0.01	-0.02	0.12	-0.04	0.00	0.01	0.06	1									
**10**	Albumin (g/dL)	0.17	0.09	-0.02	-0.08	0.14	0.04	-0.02	-0.08	-0.14	1								
**11**	Alkaline phosphatase (U/L)	-0.16	0.13	0.05	0.14	0.07	-0.03	0.09	0.13	0.06	-0.05	1							
**12**	Triglyceride (mg/dL)	0.04	0.14	0.10	0.17	0.23	0.13	0.13	0.37	-0.05	0.05	0.12	1						
**13**	Mean cell volume (fL)	-0.05	0.00	-0.03	-0.06	0.03	-0.04	-0.04	-0.13	-0.03	-0.02	-0.03	-0.15	1					
**14**	Uric acid (mg/dL)	0.22	0.19	0.28	0.09	-0.02	0.52	0.05	0.52	-0.02	0.06	0.06	0.33	-0.07	1				
**15**	Lymphocyte (%)	-0.06	-0.04	-0.04	0.03	0.10	-0.10	-0.04	-0.06	-0.05	0.04	-0.04	0.01	0.03	-0.07	1			
**16**	RBC count (million cells/µL)	0.28	0.17	0.02	0.08	0.01	0.30	0.03	0.36	0.03	0.16	0.08	0.22	-0.34	0.35	-0.09	1		
**17**	C-reactive protein (mg/dL)	-0.13	0.06	0.01	0.12	-0.02	0.00	0.07	0.21	0.10	-0.19	0.20	0.08	-0.03	0.11	-0.14	0.00	1	
**18**	DBP (mm Hg)	0.05	0.67	-0.02	0.04	0.12	0.08	0.05	0.30	0.00	0.10	0.10	0.16	-0.03	0.21	-0.02	0.26	0.07	1

Abbreviations: DBP, diastolic blood pressure; *FEV$_1$*, forced expiratory volume in 1 second; HbA1c, glycated hemoglobin; *RBC*, red blood cell; SBP, systolic blood pressure; *SD*, standard deviation.

Supplementary Table 3. Descriptive statistics for covariates in UK Biobank. Data are shown as numbers (%).

Characteristic	Total (n=308,156)	Women (n=163,022)	Men (n=145,134)
Sociodemographic and health-related factors			
Year of birth			
1930–1939	9,753 (3.2)	4,848 (3.0)	4,905 (3.4)
1940–1949	129,380 (42.0)	67,213 (41.2)	62,167 (42.8)
1950–1959	101,040 (32.8)	55,020 (33.8)	46,020 (31.7)
≥1960	67,983 (22.1)	35,941 (22.0)	32,042 (22.1)
Baseline assessment center			
England	282,887 (91.8)	149,408 (91.6)	133,479 (92.0)
Wales	13,497 (4.4)	7,198 (4.4)	6,299 (4.3)
Scotland	11,772 (3.8)	6,416 (3.9)	5,356 (3.7)
Ethnic background			
White	290,646 (94.3)	153,990 (94.5)	136,656 (94.2)
Asian	7,227 (2.3)	3,377 (2.1)	3,850 (2.7)
Black	4,281 (1.4)	2,391 (1.5)	1,890 (1.3)
Others	4,581 (1.5)	2,635 (1.6)	1,946 (1.3)
Unknown	1,421 (0.5)	629 (0.4)	792 (0.5)
Body mass index categories			
Underweight (<18.5)	1,517 (0.5)	1,209 (0.7)	308 (0.2)
Normal weight (18.5 to <25)	101,022 (32.8)	64,629 (39.6)	36,393 (25.1)
Overweight (25 to <30)	131,911 (42.8)	59,785 (36.7)	72,126 (49.7)
Obese (≥30)	73,185 (23.7)	37,197 (22.8)	35,988 (24.8)
Unknown	521 (0.2)	202 (0.1)	319 (0.2)
Education level[a]			
Low	49,839 (16.2)	26,255 (16.1)	23,584 (16.2)
Intermediate	154,193 (50.0)	83,927 (51.5)	70,266 (48.4)
High	100,641 (32.7)	51,074 (31.3)	49,567 (34.2)
Unknown	3,483 (1.1)	1,766 (1.1)	1,717 (1.2)
Deprivation index quintiles[b]			
1 (least deprived)	61,676 (20.0)	32,583 (20.0)	29,093 (20.0)
2	61,456 (19.9)	32,514 (19.9)	28,942 (19.9)
3	61,545 (20.0)	33,038 (20.3)	28,507 (19.6)
4	61,560 (20.0)	33,026 (20.3)	28,534 (19.7)
5 (most deprived)	61,551 (20.0)	31,680 (19.4)	29,871 (20.6)
Unknown	368 (0.1)	181 (0.1)	187 (0.1)
Self-reported diabetes			
No	291,863 (94.7)	156,841 (96.2)	135,022 (93.0)
Yes	15,083 (4.9)	5,575 (3.4)	9,508 (6.6)
Unknown	1,210 (0.4)	606 (0.4)	604 (0.4)
Lifestyle factors			
Smoking status			
Never	170,252 (55.2)	98,266 (60.3)	71,986 (49.6)
Previous	105,086 (34.1)	50,339 (30.9)	54,747 (37.7)
Current	31,397 (10.2)	13,673 (8.4)	17,724 (12.2)
Unknown	1,421 (0.5)	744 (0.5)	677 (0.5)
Alcohol intake frequency			
Less than 3 times a month	92,062 (29.9)	60,086 (36.9)	31,976 (22.0)
1–4 times a week	151,959 (49.3)	75,948 (46.6)	76,011 (52.4)
Daily or almost daily	63,511 (20.6)	26,670 (16.4)	36,841 (25.4)
Unknown	624 (0.2)	318 (0.2)	306 (0.2)
Physical activity level[c]			
Low	67,795 (22.0)	37,351 (22.9)	30,444 (21.0)
Moderate	117,505 (38.1)	64,829 (39.8)	52,676 (36.3)
High	112,023 (36.4)	54,546 (33.5)	57,477 (39.6)
Unknown	10,833 (3.5)	6,296 (3.9)	4,537 (3.1)
Diet			
Fresh vegetable and fruit intake[d]			
<5 portions a day	181,704 (59.0)	86,214 (52.9)	95,490 (65.8)
≥5 portions a day	121,609 (39.5)	74,795 (45.9)	46,814 (32.3)
Unknown	4,843 (1.6)	2,013 (1.2)	2,830 (1.9)

Supplementary Table 3. *(continued)*

Characteristic	Total (n=308,156)	Women (n=163,022)	Men (n=145,134)
Red meat intake[e]			
Less than twice a week	151,892 (49.3)	86,513 (53.1)	65,379 (45.0)
Twice a week or more	152,491 (49.5)	74,702 (45.8)	77,789 (53.6)
Unknown	3,773 (1.2)	1,807 (1.1)	1,966 (1.4)
Processed meat intake[f]			
Less than twice a week	210,430 (68.3)	128,699 (78.9)	81,731 (56.3)
Twice a week or more	96,681 (31.4)	33,804 (20.7)	62,877 (43.3)
Unknown	1,045 (0.3)	519 (0.3)	526 (0.4)
Women's health			
Menopausal status			
Premenopausal	-	40,616 (24.9)	-
Postmenopausal	-	97,118 (59.6)	-
Unknown	-	25,288 (15.5)	-
Hormone replacement therapy use			
Never	-	101,566 (62.3)	-
Ever	-	60,675 (37.2)	-
Unknown	-	781 (0.5)	-
Oral contraceptive use			
Never	-	29,968 (18.4)	-
Ever	-	132,363 (81.2)	-
Unknown	-	691 (0.4)	-
Parity			
0 birth	-	30,161 (18.5)	-
1–2 births	-	93,378 (57.3)	-
≥3 births	-	39,139 (24.0)	-
Unknown	-	344 (0.2)	-
Cancer screening			
Ever had breast cancer screening			
No	-	34,649 (21.3)	-
Yes	-	127,906 (78.5)	-
Unknown	-	467 (0.3)	-
Ever had prostate specific antigen test			
No	-	-	97,767 (67.4)
Yes	-	-	39,643 (27.3)
Unknown	-	-	7,724 (5.3)
Ever had colorectal cancer screening			
No	210,535 (68.3)	113,695 (69.7)	96,840 (66.7)
Yes	92,146 (29.9)	47,323 (29.0)	44,823 (30.9)
Unknown	5,475 (1.8)	2,004 (1.2)	3,471 (2.4)
Family history of cancers[g]			
Family history of breast cancer	31,569 (10.2)	17,379 (10.7)	14,190 (9.8)
Family history of prostate cancer	23,447 (7.6)	12,529 (7.7)	10,918 (7.5)
Family history of lung cancer	37,502 (12.2)	20,227 (12.4)	17,275 (11.9)
Family history of colorectal cancer	33,054 (10.7)	17,155 (10.5)	15,899 (11.0)
Melanoma-related factors			
Time spent outdoors during summer[h]			
<1h/day	12,743 (4.1)	7,712 (4.7)	5,031 (3.5)
1–2h/day	90,980 (29.5)	50,944 (31.2)	40,036 (27.6)
3–5h/day	127,731 (41.5)	70,714 (43.4)	57,017 (39.3)
>5h/day	59,179 (19.2)	22,668 (13.9)	36,511 (25.2)
Unknown	17,523 (5.7)	10,984 (6.7)	6,539 (4.5)
Sunburn during childhood[i]			
No	125,359 (40.7)	71,998 (44.2)	53,361 (36.8)
Yes	107,068 (34.7)	52,557 (32.2)	54,511 (37.6)
Unknown	75,729 (24.6)	38,467 (23.6)	37,262 (25.7)
Solarium/sunlamp use[j]			
No	290,812 (94.4)	151,870 (93.2)	138,942 (95.7)
Yes	13,692 (4.4)	9,182 (5.6)	4,510 (3.1)
Unknown	3,652 (1.2)	1,970 (1.2)	1,682 (1.2)

Supplementary Table 3. *(continued)*

Characteristic	Total (n=308,156)	Women (n=163,022)	Men (n=145,134)
Ease of skin tanning[k]			
Get very tanned	65,023 (21.1)	27,299 (16.7)	37,724 (26.0)
Get moderately tanned	120,635 (39.1)	61,591 (37.8)	59,044 (40.7)
Get mildly or occasionally tanned	63,407 (20.6)	38,865 (23.8)	24,542 (16.9)
Never tan, only burn	50,531 (16.4)	30,513 (18.7)	20,018 (13.8)
Unknown	8,560 (2.8)	4,754 (2.9)	3,806 (2.6)
Skin color[l]			
Black, brown	10,969 (3.6)	4,924 (3.0)	6,045 (4.2)
Light, dark olive	62,787 (20.4)	34,107 (20.9)	28,680 (19.8)
Fair	206,721 (67.1)	107,774 (66.1)	98,947 (68.2)
Very fair	23,023 (7.5)	14,172 (8.7)	8,851 (6.1)
Unknown	4,656 (1.5)	2,045 (1.3)	2,611 (1.8)
Hair color[m]			
Black, dark brown, other	143,622 (46.6)	70,928 (43.5)	72,694 (50.1)
Light brown	118,170 (38.3)	64,779 (39.7)	53,391 (36.8)
Blonde, red	45,358 (14.7)	26,971 (16.5)	18,387 (12.7)
Unknown	1,006 (0.3)	344 (0.2)	662 (0.5)
Use of sun/UV protection[n]			
Never/rarely	31,170 (10.1)	8,963 (5.5)	22,207 (15.3)
Sometimes	103,488 (33.6)	45,335 (27.8)	58,153 (40.1)
Most of the time	108,987 (35.4)	64,405 (39.5)	44,582 (30.7)
Always	61,810 (20.1)	42,876 (26.3)	18,934 (13.0)
Do not go out in sunshine	1,757 (0.6)	1,037 (0.6)	720 (0.5)
Unknown	944 (0.3)	406 (0.2)	538 (0.4)

[a] Education was assessed by the highest self-reported qualification: low (no relevant qualifications); intermediate (A levels, O levels/GCSEs, CSEs, NVQ/HND/HNC, other professional qualifications); high (college or university degree).
[b] Townsend deprivation index was derived from national census data regarding unemployment, car ownership, home ownership, and household overcrowding. A higher score indicates a higher level of socioeconomic deprivation.
[c] Physical activity was assessed by the self-reported International Physical Activity Questionnaire (IPAQ) and was categorized into low, moderate, high according to the protocol.
[d] Fresh vegetable and fruit intake was assessed by the questions "on average how many heaped tablespoons of salad or raw vegetables would you eat per day? ", and "about how many pieces of fresh fruit would you eat per day?"
[e] Red meat intake was assessed by the questions "how often do you eat beef?", "how often do you eat lamb/mutton?", and "how often do you eat pork?"
[f] Processed meat intake was assessed by the question "how often do you eat processed meats (such as bacon, ham, sausages, meat pies, kebabs, burgers, chicken nuggets)?"
[g] Family history of cancer was defined by any self-reported cancers in father, mother or siblings.
[h] Time spent outdoors during summer was assessed by the question "in a typical day in summer, how many hours do you spend outdoors?"
[i] Childhood sunburns were assessed by the question "before the age of 15, how many times did you suffer sunburn that was painful for at least 2 days or caused blistering?"
[j] Solarium/sunlamp use was assessed by the question "how many times a year would you use a solarium or sunlamp?", and was categorized into no (0 time a year) and yes (1 or more time a year).
[k] Ease of skin tanning was assessed by the question "what would happen to your skin if it was repeatedly exposed to bright sunlight without any protection?"
[l] Skin color was assessed by the question "what best describes the color of your skin without tanning?"
[m] Hair color was assessed by the question "what best describes your natural hair color? (If your hair color is grey, the color before you went grey)"
[n] Use of UV protection was assessed by the question "do you wear sun protection (e.g. sunscreen lotion, hat) when you spend time outdoors in the summer?"

Supplementary Table 4. Associations between individual clinical biomarkers and risk of cancer in UK Biobank (n=308,156)

Biomarker	Any cancer		Breast cancer in women		Prostate cancer in men		Lung cancer		Colorectal cancer		Melanoma	
	HR (95% CI)	*p*	HR (95% CI)	*p*	HR (95% CI)	*p*	HR (95% CI)	*p*	HR (95% CI)	*p*	HR (95% CI)	*p*
FEV1	0.98 (0.96, 0.99)	0.001	1.00 (0.96, 1.05)	0.884	1.05 (1.02, 1.08)	0.001	0.63 (0.60, 0.67)	<.001	0.98 (0.94, 1.02)	0.363	1.10 (1.03, 1.17)	0.005
SBP	1.00 (0.99, 1.02)	0.479	1.05 (1.02, 1.07)	0.001	1.01 (0.99, 1.04)	0.391	1.00 (0.96, 1.04)	0.883	1.04 (1.01, 1.08)	0.014	0.99 (0.94, 1.04)	0.579
Blood urea nitrogen	0.99 (0.98, 1.00)	0.078	0.96 (0.93, 0.99)	0.005	1.02 (1.00, 1.05)	0.059	0.93 (0.90, 0.97)	0.001	0.95 (0.92, 0.98)	0.003	1.00 (0.96, 1.05)	0.879
HbA1c	1.01 (1.00, 1.02)	0.015	0.98 (0.95, 1.01)	0.164	0.96 (0.94, 0.99)	0.013	1.06 (1.03, 1.10)	0.001	1.03 (1.00, 1.06)	0.058	0.99 (0.93, 1.04)	0.575
Total cholesterol	0.94 (0.93, 0.95)	<.001	0.98 (0.96, 1.01)	0.226	0.99 (0.97, 1.01)	0.424	0.90 (0.86, 0.93)	<.001	0.99 (0.96, 1.03)	0.675	0.98 (0.94, 1.03)	0.476
Creatinine	1.02 (1.01, 1.04)	<.001	1.01 (0.97, 1.05)	0.597	1.01 (0.99, 1.04)	0.424	0.99 (0.95, 1.04)	0.676	0.99 (0.95, 1.03)	0.566	1.08 (1.03, 1.14)	0.002
Serum glucose	1.01 (1.00, 1.02)	0.022	1.00 (0.97, 1.03)	0.972	0.97 (0.94, 0.99)	0.014	1.01 (0.97, 1.05)	0.688	1.05 (1.02, 1.08)	0.001	1.00 (0.95, 1.05)	0.981
Waist circumference	1.11 (1.09, 1.13)	<.001	1.11 (1.06, 1.16)	<.001	1.01 (0.97, 1.06)	0.618	1.16 (1.08, 1.24)	<.001	1.15 (1.08, 1.21)	<.001	0.99 (0.91, 1.08)	0.855
Red cell distribution width	1.09 (1.08, 1.10)	<.001	1.02 (0.99, 1.04)	0.126	1.00 (0.97, 1.03)	0.942	1.12 (1.08, 1.16)	<.001	1.08 (1.05, 1.12)	<.001	1.06 (1.02, 1.11)	0.009
Albumin	0.94 (0.93, 0.95)	<.001	0.97 (0.95, 1.00)	0.042	1.01 (0.98, 1.03)	0.589	0.90 (0.86, 0.93)	<.001	0.92 (0.89, 0.95)	<.001	0.99 (0.94, 1.04)	0.589
Alkaline phosphatase	1.01 (1.00, 1.02)	0.031	0.97 (0.94, 1.00)	0.023	0.99 (0.96, 1.02)	0.429	1.11 (1.08, 1.14)	<.001	1.04 (1.00, 1.07)	0.028	0.96 (0.91, 1.01)	0.080
Triglyceride	0.98 (0.97, 0.99)	<.001	0.99 (0.95, 1.02)	0.384	0.97 (0.95, 1.00)	0.020	1.01 (0.97, 1.05)	0.667	1.03 (1.00, 1.07)	0.078	0.96 (0.92, 1.02)	0.170
Mean cell volume	1.00 (0.99, 1.01)	0.830	1.00 (0.97, 1.03)	0.954	0.96 (0.93, 0.98)	<.001	1.13 (1.09, 1.18)	<.001	0.99 (0.96, 1.02)	0.533	0.97 (0.92, 1.01)	0.169
Uric acid	1.01 (1.00, 1.02)	0.093	1.02 (0.98, 1.05)	0.310	0.99 (0.97, 1.02)	0.595	1.02 (0.97, 1.07)	0.370	1.00 (0.96, 1.04)	0.846	1.01 (0.95, 1.07)	0.819
Lymphocyte	0.97 (0.96, 0.98)	<.001	0.97 (0.94, 1.00)	0.030	0.99 (0.97, 1.02)	0.616	0.93 (0.89, 0.97)	0.001	0.94 (0.91, 0.98)	0.001	1.00 (0.95, 1.04)	0.880
RBC count	0.97 (0.96, 0.99)	<.001	1.06 (1.02, 1.09)	0.001	1.05 (1.02, 1.08)	0.001	0.98 (0.93, 1.03)	0.371	0.94 (0.90, 0.97)	0.001	1.01 (0.96, 1.07)	0.622
C-reactive protein	1.04 (1.03, 1.05)	<.001	1.02 (0.99, 1.05)	0.131	0.98 (0.96, 1.01)	0.152	1.11 (1.08, 1.14)	<.001	1.07 (1.04, 1.10)	<.001	0.99 (0.95, 1.04)	0.821
DBP	1.00 (0.99, 1.02)	0.378	1.04 (1.02, 1.07)	0.002	1.03 (1.00, 1.05)	0.034	0.94 (0.90, 0.98)	0.005	1.03 (0.99, 1.06)	0.149	0.99 (0.94, 1.04)	0.703

Notes: *DBP*, diastolic blood pressure; *FEV_1*, forced expiratory volume in 1 second; *HbA1c*, glycated hemoglobin; *RBC*, red blood cell; *SBP*, systolic blood pressure; *WBC*, white blood cell. All biomarkers were standardized with mean=0 and standard deviation=1, and HRs were standard deviation increase in the biomarker level. All models were adjusted for age (time scale), birth year, sex, baseline assessment center, ethnic background, body mass index, smoking status, physical activity level, alcohol consumption, education level, deprivation index quintiles, and the cancer-specific covariates as detailed in the Supplementary Table 3 and the footnote of Table 3.

Supplementary Table 5. Subgroup analyses for the associations between biological age measures and risk of cancer in UK Biobank[a]

Cancer site	KDM residual		PhenoAge residual		HD (log)	
	HR per 1 SD increase (95% CI)	$P_{interaction}$[b]	HR per 1 SD increase (95% CI)	$P_{interaction}$[b]	HR per 1 SD increase (95% CI)	$P_{interaction}$[b]
**Any cancer**						
Age at baseline		0.41		0.12		0.14
<60 years (n = 179,827)	1.03 (1.01, 1.05)*		1.10 (1.08, 1.12)*		1.02 (1.00, 1.04)	
≥60 years (n = 128,329)	1.04 (1.03, 1.06)*		1.07 (1.05, 1.08)*		1.04 (1.03, 1.06)*	
Sex		0.13		0.012		<.001
Women (n = 163,022)	1.02 (1.01, 1.04)		1.09 (1.07, 1.11)*		1.05 (1.03, 1.07)*	
Men (n = 145,134)	1.06 (1.04, 1.07)*		1.06 (1.05, 1.08)*		1.04 (1.02, 1.05)*	
Ethnicity		0.10		0.11		0.046
White (n = 290,646)	1.04 (1.03, 1.05)*		1.09 (1.08, 1.10)*		1.02 (1.01, 1.03)*	
Non-white (n = 16,089)	1.00 (0.94, 1.06)		1.05 (1.00, 1.11)*		0.96 (0.90, 1.02)	
**Breast cancer in women**						
Age at baseline		0.17		0.18		0.96
<60 years (n = 96,792)	1.00 (0.97, 1.05)		1.05 (1.01, 1.09)		1.02 (0.98, 1.06)	
≥60 years (n = 66,230)	1.02 (0.98, 1.06)		1.06 (1.02, 1.11)		0.99 (0.94, 1.04)	
Menopausal status		<.001		0.002		0.09
Premenopausal (n = 40,616)	0.99 (0.92, 1.05)		1.01 (0.95, 1.08)		1.03 (0.96, 1.09)	
Postmenopausal (n = 97,118)	1.03 (0.99, 1.07)		1.08 (1.04, 1.12)*		1.01 (0.97, 1.05)	
Ethnicity		0.81		0.32		0.71
White (n = 153,990)	1.01 (0.98, 1.04)		1.06 (1.03, 1.09)*		1.01 (0.98, 1.04)	
Non-white (n = 8,403)	1.01 (0.88, 1.17)		1.01 (0.89, 1.15)		0.98 (0.84, 1.14)	
**Prostate cancer in men**						
Age at baseline		0.40		0.21		0.74
<60 years (n =83,035)	0.99 (0.94, 1.04)		1.00 (0.95, 1.04)		0.98 (0.94, 1.03)	
≥60 years (n = 62,099)	0.95 (0.92, 0.98)*		0.95 (0.92, 0.98)*		0.97 (0.94, 1.00)	
Ethnicity		0.07		<.001		0.20
White (n = 136,656)	0.96 (0.93, 0.98)*		0.96 (0.94, 0.98)*		0.97 (0.95, 1.00)	
Non-white (n = 7,686)	1.01 (0.89, 1.14)		1.04 (0.93, 1.16)		0.99 (0.87, 1.13)	
**Lung cancer**						
Age at baseline		0.88		0.64		0.94
<60 years (n = 179,827)	1.32 (1.22, 1.42)*		1.37 (1.28, 1.46)*		1.15 (1.07, 1.24)*	
≥60 years (n = 128,329)	1.28 (1.22, 1.35)*		1.34 (1.28, 1.40)*		1.11 (1.06, 1.18)*	
Sex		0.050		0.29		0.81
Women (n = 163,022)	1.24 (1.16, 1.32)*		1.33 (1.25, 1.41)*		1.14 (1.07, 1.22)*	
Men (n = 145,134)	1.34 (1.27, 1.41)*		1.37 (1.30, 1.44)*		1.12 (1.06, 1.19)*	
Smoking		<.001		<.001		<.001
Never-smoker (n = 170,252)	0.92 (0.82, 1.04)		0.97 (0.86, 1.09)		0.91 (0.80, 1.03)	
Ever-smoker (n = 136,483)	1.42 (1.36, 1.48)*		1.51 (1.45, 1.56)*		1.20 (1.14, 1.25)*	
Ethnicity		0.001		0.003		0.011
White (n = 290,646)	1.30 (1.25, 1.36)*		1.36 (1.31, 1.42)*		1.13 (1.08, 1.18)*	
Non-white (n = 16,089)	0.93 (0.72, 1.20)		1.05 (0.83, 1.31)		0.90 (0.69, 1.18)	
**Colorectal cancer**						
Age at baseline		0.11		0.47		0.29
<60 years (n = 179,827)	1.13 (1.06, 1.20)*		1.10 (1.04, 1.17)*		1.13 (1.07, 1.20)*	
≥60 years (n = 128,329)	1.06 (1.02, 1.11)		1.07 (1.03, 1.12)*		1.09 (1.04, 1.14)*	
Sex		0.001		0.032		0.39
Women (n = 163,022)	1.04 (0.98, 1.10)		1.05 (0.99, 1.10)		1.12 (1.06, 1.19)*	
Men (n = 145,134)	1.13 (1.08, 1.18)*		1.11 (1.06, 1.16)*		1.10 (1.05, 1.15)*	
Ethnicity		0.48		0.71		0.62
White (n = 290,646)	1.09 (1.05, 1.13)*		1.08 (1.05, 1.12)*		1.11 (1.07, 1.15)*	
Non-white (n = 16,089)	1.02 (0.84, 1.24)		1.08 (0.91, 1.28)		1.04 (0.85, 1.28)	

Supplementary Table 5. *(continued)*

Cancer site	KDM residual		PhenoAge residual		HD (log)	
	HR per 1 SD increase (95% CI)	$P_{interaction}$[b]	HR per 1 SD increase (95% CI)	$P_{interaction}$[b]	HR per 1 SD increase (95% CI)	$P_{interaction}$[b]
**Melanoma**						
Age at baseline		<.001		0.005		0.17
<60 years (n = 179,827)	0.86 (0.80, 0.94)*		0.95 (0.88, 1.02)		0.92 (0.85, 0.99)	
≥60 years (n = 128,329)	1.03 (0.96, 1.10)		1.05 (0.99, 1.13)		1.02 (0.94, 1.09)	
Sex		0.09		0.81		0.017
Women (n = 163,022)	0.93 (0.87, 1.01)		1.03 (0.95, 1.11)		0.96 (0.89, 1.04)	
Men (n = 145,134)	0.98 (0.91, 1.05)		0.99 (0.92, 1.06)		0.98 (0.91, 1.05)	
Ethnicity		0.90		0.99		0.57
White (n = 290,646)	0.95 (0.90, 1.00)		1.01 (0.96, 1.06)		0.95 (0.90, 1.01)	
Non-white (n = 16,089)	1.07 (0.46, 2.50)		1.18 (0.58, 2.41)		1.32 (0.63, 2.75)	

Abbreviations: *HD*, homeostatic dysregulation; *HR*, hazard ratio; *KDM*, Klemera-Doubal method; *SD*, standard deviation.

[a] Models were adjusted for age, birth year, sex, baseline assessment center, ethnic background, body mass index, smoking status, alcohol consumption, physical activity level, education level, and deprivation index quintiles, and the cancer-specific covariates, except when the variable was used as the subgroup.

[b] *P*-values for the multiplicative interaction terms between the continuous biological age measures and the subgroup indicator.

* Significant after Bonferroni correction at p<.05/15 (i.e., 5 cancers × 3 biological age measures)

Supplementary Table 6. Associations between biological age measures using the Levine original KDM and PhenoAge algorithms and risk of cancer in UK Biobank[a]

Cancer site	**Levine original KDM residual**		**Levine original PhenoAge residual**	
	HR per 1 SD increase (95% CI)	*p*	HR per 1 SD increase (95% CI)	*p*
**Any cancer**				
Multivariable model[b]	1.02 (1.01, 1.03)*	0.001	1.12 (1.11, 1.13)*	<.001
**Breast cancer in women**				
Breast cancer-specific model[c]	1.00 (0.97, 1.03)	0.89	1.04 (1.02, 1.07)*	0.002
**Prostate cancer in men**				
Prostate cancer-specific model[d]	0.98 (0.96, 1.00)	0.044	0.98 (0.95, 1.00)	0.08
**Lung cancer**				
Lung cancer-specific model[e]	1.26 (1.21, 1.32)*	<.001	1.25 (1.21, 1.30)*	<.001
**Colorectal cancer**				
Colorectal cancer-specific model[f]	1.05 (1.01, 1.08)	0.008	1.12 (1.08, 1.16)*	<.001
**Melanoma**				
Melanoma-specific model[g]	0.96 (0.91, 1.01)	0.11	1.05 (1.00, 1.11)	0.040

Abbreviations: CI, confidence interval; *HR*, hazard ratio; KDM, Klemera-Doubal method; *SD*, standard deviation.

[a] As shown in Table 1, we used the original list of biomarkers included in Levine 2013 and Levine et al. 2018 to calculate the "Levine original KDM" and "Levine original PhenoAge", respectively.

[b] Multivariable model: adjusted for age (time scale), birth year (1930–1939, 1940–1949, 1950–1959, ≥1960), sex, baseline assessment center (England, Wales, Scotland), ethnic background (White, Asian, Black, others), body mass index (underweight, normal weight, overweight, obese), smoking status (never, previous, current), physical activity level (low, moderate, high), alcohol consumption (less than 3 times a month, 1–4 times a week, daily or almost daily), education level (high, intermediate, low), deprivation index quintiles (1st, 2nd, 3rd, 4th, 5th).

[c] Breast cancer-specific model: multivariable model + family history of breast cancer (no, yes), ever had breast cancer screening (no, yes), menopause (premenopausal, postmenopausal), hormone replacement therapy use (never, ever), oral contraceptive use (never, ever), parity (0, 1–2, ≥3).

[d] Prostate cancer-specific model: multivariable model + family history of prostate cancer (no, yes), ever had prostate specific antigen test (no, yes), self-reported diabetes (no, yes).

[e] Lung cancer-specific model: multivariable model + family history of lung cancer (no, yes).

[f] Colorectal cancer-specific model: multivariable model + family history of colorectal cancer (no, yes), ever had colorectal cancer screening (no, yes), fresh vegetable and fruit intake (<5 portions a day, ≥5 portions a day), red meat intake (less than twice a week, twice a week or more), processed meat intake (less than twice a week, twice a week or more).

[g] Melanoma cancer-specific model: multivariable model + time spent outdoors during summer (1–2h/day, 3–5h/day, >5h/day), use of sun/UV protection (never/rarely, sometimes, most of the time, always, do not go out in sunshine), sunburn during childhood (no, yes), solarium/sunlamp use (no, yes), ease of skin tanning (very tanned, moderately tanned, mildly or occasionally tanned, never tan but only burn), skin color (black/brown, light/dark olive, fair, very fair), hair color (black/dark brown/other, light brown, blonde/red).

* Significant after Bonferroni correction at *p*<.05/15 (i.e., 5 cancers × 3 biological age measures)

Supplementary Table 7. Associations between modified biological age measures excluding HbA1c and serum glucose from the algorithms and risk of cancer in UK Biobank[a]

Cancer site	Modified KDM residual		Modified PhenoAge residual		Modified HD (log)	
	HR per 1 SD increase (95% CI)	*p*	HR per 1 SD increase (95% CI)	*p*	HR per 1 SD increase (95% CI)	*p*
**Any cancer**						
Multivariable model[b]	1.03 (1.02, 1.04)*	<.001	1.09 (1.08, 1.10)*	<.001	1.02 (1.01, 1.03)	0.004
**Breast cancer in women**						
Breast cancer-specific model[c]	1.01 (0.98, 1.04)	0.42	1.06 (1.03, 1.09)*	<.001	1.00 (0.97, 1.03)	0.87
**Prostate cancer in men**						
Prostate cancer-specific model[d]	0.96 (0.94, 0.99)	0.008	0.97 (0.95, 1.00)	0.020	0.98 (0.95, 1.00)	0.06
**Lung cancer**						
Lung cancer-specific model[e]	1.29 (1.24, 1.35)*	<.001	1.36 (1.31, 1.41)*	<.001	1.13 (1.08, 1.18)*	<.001
**Colorectal cancer**						
Colorectal cancer-specific model[f]	1.07 (1.04, 1.11)*	<.001	1.08 (1.05, 1.12)*	<.001	1.11 (1.07, 1.15)*	<.001
**Melanoma**						
Melanoma-specific model[g]	0.95 (0.90, 1.00)	0.047	1.01 (0.96, 1.06)	0.68	0.95 (0.90, 1.00)	0.04

Abbreviations: CI, confidence interval; *HD*, homeostatic dysregulation; *HR*, hazard ratio; *KDM*, Klemera-Doubal method; *SD*, standard deviation.

[a] We included 16 biomarkers in the modified algorithms of KDM, PhenoAge, and HD (excluding HbA1c and serum glucose) to test whether these two items would affect the results. List of the included biomarkers is shown in Table 1.

[b] Multivariable model: adjusted for age (time scale), birth year (1930–1939, 1940–1949, 1950–1959, ≥1960), sex, baseline assessment center (England, Wales, Scotland), ethnic background (White, Asian, Black, others), body mass index (underweight, normal weight, overweight, obese), smoking status (never, previous, current), physical activity level (low, moderate, high), alcohol consumption (less than 3 times a month, 1–4 times a week, daily or almost daily), education level (high, intermediate, low), deprivation index quintiles (1st, 2nd, 3rd, 4th, 5th).

[c] Breast cancer-specific model: multivariable model + family history of breast cancer (no, yes), ever had breast cancer screening (no, yes), menopause (premenopausal, postmenopausal), hormone replacement therapy use (never, ever), oral contraceptive use (never, ever), parity (0, 1–2, ≥3).

[d] Prostate cancer-specific model: multivariable model + family history of prostate cancer (no, yes), ever had prostate specific antigen test (no, yes), self-reported diabetes (no, yes).

[e] Lung cancer-specific model: multivariable model + family history of lung cancer (no, yes).

[f] Colorectal cancer-specific model: multivariable model + family history of colorectal cancer (no, yes), ever had colorectal cancer screening (no, yes), fresh vegetable and fruit intake (<5 portions a day, ≥5 portions a day), red meat intake (less than twice a week, twice a week or more), processed meat intake (less than twice a week, twice a week or more).

[g] Melanoma cancer-specific model: multivariable model + time spent outdoors during summer (1–2h/day, 3–5h/day, >5h/day), use of sun/UV protection (never/rarely, sometimes, most of the time, always, do not go out in sunshine), sunburn during childhood (no, yes), solarium/sunlamp use (no, yes), ease of skin tanning (very tanned, moderately tanned, mildly or occasionally tanned, never tan but only burn), skin color (black/brown, light/dark olive, fair, very fair), hair color (black/dark brown/other, light brown, blonde/red).

* Significant after Bonferroni correction at *p*<.05/15 (i.e., 5 cancers × 3 biological age measures)

Supplementary Table 8. Associations between biological age measures and risk of cancer in using complete data in UK Biobank[a]

Cancer site	KDM residual		PhenoAge residual		HD (log)	
	HR per 1 SD increase (95% CI)	*p*	HR per 1 SD increase (95% CI)	*p*	HR per 1 SD increase (95% CI)	*p*
**Any cancer**						
Multivariable model (n = 291,898)[b]	1.04 (1.02, 1.05)*	<.001	1.09 (1.07, 1.10)*	<.001	1.02 (1.01, 1.03)*	0.003
**Breast cancer in women**						
Breast cancer-specific model (n = 129,944)[c]	1.01 (0.98, 1.05)	0.42	1.06 (1.03, 1.09)*	<.001	1.00 (0.97, 1.04)	0.84
**Prostate cancer in men**						
Prostate cancer-specific model (n = 130,598)[d]	0.96 (0.94, 0.99)	0.004	0.97 (0.94, 0.99)	0.008	0.97 (0.95, 1.00)	0.06
**Lung cancer**						
Lung cancer-specific model (n = 291,898)[e]	1.28 (1.22, 1.33)*	<.001	1.34 (1.29, 1.40)*	<.001	1.11 (1.06, 1.16)*	<.001
**Colorectal cancer**						
Colorectal cancer-specific model (n = 282,292)[f]	1.09 (1.05, 1.13)*	<.001	1.08 (1.05, 1.12)*	<.001	1.10 (1.06, 1.15)*	<.001
**Melanoma**						
Melanoma-specific model (n = 207,355)[g]	0.93 (0.87, 0.99)	0.034	1.00 (0.94, 1.06)	0.95	0.96 (0.90, 1.02)	0.18

Abbreviations: CI, confidence interval; *HD*, homeostatic dysregulation; *HR*, hazard ratio; *KDM*, Klemera-Doubal method; *SD*, standard deviation.

[a] Individuals with missing data on any covariate were excluded.

[b] Multivariable model: adjusted for age (time scale), birth year (1930–1939, 1940–1949, 1950–1959, ≥1960), sex, baseline assessment center (England, Wales, Scotland), ethnic background (White, Asian, Black, others), body mass index (underweight, normal weight, overweight, obese), smoking status (never, previous, current), physical activity level (low, moderate, high), alcohol consumption (less than 3 times a month, 1–4 times a week, daily or almost daily), education level (high, intermediate, low), deprivation index quintiles (1st, 2nd, 3rd, 4th, 5th).

[c] Breast cancer-specific model: multivariable model + family history of breast cancer (no, yes), ever had breast cancer screening (no, yes), menopause (premenopausal, postmenopausal), hormone replacement therapy use (never, ever), oral contraceptive use (never, ever), parity (0, 1–2, ≥3).

[d] Prostate cancer-specific model: multivariable model + family history of prostate cancer (no, yes), ever had prostate specific antigen test (no, yes), self-reported diabetes (no, yes).

[e] Lung cancer-specific model: multivariable model + family history of lung cancer (no, yes).

[f] Colorectal cancer-specific model: multivariable model + family history of colorectal cancer (no, yes), ever had colorectal cancer screening (no, yes), fresh vegetable and fruit intake (<5 portions a day, ≥5 portions a day), red meat intake (less than twice a week, twice a week or more), processed meat intake (less than twice a week, twice a week or more).

[g] Melanoma cancer-specific model: multivariable model + time spent outdoors during summer (1–2h/day, 3–5h/day, >5h/day), use of sun/UV protection (never/rarely, sometimes, most of the time, always, do not go out in sunshine), sunburn during childhood (no, yes), solarium/sunlamp use (no, yes), ease of skin tanning (very tanned, moderately tanned, mildly or occasionally tanned, never tan but only burn), skin color (black/brown, light/dark olive, fair, very fair), hair color (black/dark brown/other, light brown, blonde/red).

* Significant after Bonferroni correction at *p*<.05/15 (i.e., 5 cancers × 3 biological age measures)

Supplementary Table 9. Associations between biological age measures and risk of cancer excluding individuals with <2 years follow-up

Cancer site	KDM residual		PhenoAge residual		HD (log)	
	HR per 1 SD increase (95% CI)	*p*	HR per 1 SD increase (95% CI)	*p*	HR per 1 SD increase (95% CI)	*p*
**Any cancer**						
Multivariable model (n = 302,342)[a]	1.03 (1.02, 1.05)*	<.001	1.08 (1.07, 1.09)*	<.001	1.00 (0.99, 1.02)	0.56
**Breast cancer in women**						
Breast cancer-specific model (n = 160,192)[b]	1.02 (0.99, 1.05)	0.21	1.06 (1.02, 1.09)*	0.001	0.98 (0.95, 1.02)	0.32
**Prostate cancer in men**						
Prostate cancer-specific model (n = 142,150)[c]	0.96 (0.93, 0.99)	0.005	0.96 (0.94, 0.99)	0.005	0.97 (9.94, 1.00)	0.030
**Lung cancer**						
Lung cancer-specific model (n = 302,342)[d]	1.29 (1.23, 1.34)*	<.001	1.33 (1.28, 1.39)*	<.001	1.11 (1.06, 1.16)*	<.001
**Colorectal cancer**						
Colorectal cancer-specific model (n = 302,342)[e]	1.08 (1.04, 1.12)*	<.001	1.06 (1.02, 1.10)*	0.002	1.09 (1.05, 1.14)*	<.001
**Melanoma**						
Melanoma-specific model (n = 302,342)[f]	0.94 (0.89, 1.00)	0.041	1.01 (0.95, 1.06)	0.86	0.95 (0.89, 1.00)	0.07

Abbreviations: CI, confidence interval; *HD*, homeostatic dysregulation; *HR*, hazard ratio; *KDM*, Klemera-Doubal method; *SD*, standard deviation.

[a] Multivariable model: adjusted for age (time scale), birth year (1930–1939, 1940–1949, 1950–1959, ≥1960), sex, baseline assessment center (England, Wales, Scotland), ethnic background (White, Asian, Black, others), body mass index (underweight, normal weight, overweight, obese), smoking status (never, previous, current), physical activity level (low, moderate, high), alcohol consumption (less than 3 times a month, 1–4 times a week, daily or almost daily), education level (high, intermediate, low), deprivation index quintiles (1st, 2nd, 3rd, 4th, 5th).

[b] Breast cancer-specific model: multivariable model + family history of breast cancer (no, yes), ever had breast cancer screening (no, yes), menopause (premenopausal, postmenopausal), hormone replacement therapy use (never, ever), oral contraceptive use (never, ever), parity (0, 1–2, ≥3).

[c] Prostate cancer-specific model: multivariable model + family history of prostate cancer (no, yes), ever had prostate specific antigen test (no, yes), self-reported diabetes (no, yes).

[d] Lung cancer-specific model: multivariable model + family history of lung cancer (no, yes).

[e] Colorectal cancer-specific model: multivariable model + family history of colorectal cancer (no, yes), ever had colorectal cancer screening (no, yes), fresh vegetable and fruit intake (<5 portions a day, ≥5 portions a day), red meat intake (less than twice a week, twice a week or more), processed meat intake (less than twice a week, twice a week or more).

[f] Melanoma cancer-specific model: multivariable model + time spent outdoors during summer (1–2h/day, 3–5h/day, >5h/day), use of sun/UV protection (never/rarely, sometimes, most of the time, always, do not go out in sunshine), sunburn during childhood (no, yes), solarium/sunlamp use (no, yes), ease of skin tanning (very tanned, moderately tanned, mildly or occasionally tanned, never tan but only burn), skin color (black/brown, light/dark olive, fair, very fair), hair color (black/dark brown/other, light brown, blonde/red).

* Significant after Bonferroni correction at p<.05/15 (i.e., 5 cancers × 3 biological age measures)
